# Cyclotides Isolated From Violet Plants of Cameroon Are Inhibitors of Human Prolyl Oligopeptidase

**DOI:** 10.3389/fphar.2021.707596

**Published:** 2021-07-12

**Authors:** Jasmin Gattringer, Olivier Eteme Ndogo, Bernhard Retzl, Carina Ebermann, Christian W. Gruber, Roland Hellinger

**Affiliations:** Center for Physiology and Pharmacology, Medical University of Vienna, Vienna, Austria

**Keywords:** cyclic cystine-rich peptides, prolyl oligopeptidase, Allexis cauliflora, peptide, protease inhibitor

## Abstract

Traditional medicine and the use of herbal remedies are well established in the African health care system. For instance, Violaceae plants are used for antimicrobial or anti-inflammatory applications in folk medicine. This study describes the phytochemical analysis and bioactivity screening of four species of the violet *tribe* Allexis found in Cameroon. *Allexis cauliflora*, *Allexis obanensis*, *Allexis batangae* and *Allexis zygomorpha* were evaluated for the expression of circular peptides (cyclotides) by mass spectrometry. The unique cyclic cystine-rich motif was identified in several peptides of all four species. Knowing that members of this peptide family are protease inhibitors, the plant extracts were evaluated for the inhibition of human prolyl oligopeptidase (POP). Since all four species inhibited POP activity, a bioactivity-guided fractionation approach was performed to isolate peptide inhibitors. These novel cyclotides, alca 1 and alca 2 exhibited IC_50_ values of 8.5 and 4.4 µM, respectively. To obtain their amino acid sequence information, combinatorial enzymatic proteolysis was performed. The proteolytic fragments were evaluated in MS/MS fragmentation experiments and the full-length amino acid sequences were obtained by *de novo* annotation of fragment ions. In summary, this study identified inhibitors of the human protease POP, which is a drug target for inflammatory or neurodegenerative disorders.

## Introduction

In Cameroon, like in many other African countries, about 80% of the population relies on traditional medicine and uses herbal remedies as health supplements and to treat diseases (Njamen et al., 2013). Of the plants documented in the Cameroonian Pharmacopoeia, Violaceae are used in various medicinal applications. For example, *Viola odorata* L. has traditional applications to treat anxiety (Mousavi et al., 2016), insomnia (Feyzabadi et al., 2014), and hypertension (Siddiqi et al., 2012; Mousavi et al., 2016); *Hybanthus enneaspermus* (L.) F. Muell. is being used as an aphrodisiac, demulcent, tonic and diuretic (Weniger et al., 2004; Patel et al., 2011); *Rinorea dentata* (P.Beauv.) Kuntze is being used for treatment of neurodegenerative disorders (Sonibare and Ayoola, 2015); and other *Rinorea* species are reportedly used to treat infections (such as malaria, HIV, and syphilis) or to mitigate abdominal pain (Munvera et al., 2020). However, herbal preparations of these plants have been mainly used at a traditional level and their beneficial effects have not been determined in evidence-based clinical studies, yet. Therefore, it can be useful to isolate and characterize the active molecule(s) of these plants to trigger detailed preclinical or clinical studies in the future (Atanasov et al., 2021).

Violaceae consists of approximately 1,100 species of herbs, shrubs, lianas, bushes and trees (Park et al., 2017), and many of them grow in the sub- and tropic areas of the African continent. The genus *Allexis* within Violaceae comprises only four species, i.e*. Allexis batangae* (Engl.) Melch, *Allexis cauliflora* (Oliv.) Pierre, *Allexis obanensis* (Baker f.) Melch and *Allexis zygomorpha* Achound. & Onana; they are commonly distributed from Congo to Cameroon. Antimicrobial flavonoids and triterpene were previously isolated from Allexis species highlighting the benefit of bioactive molecules in phytotherapy, but these plants have not been previously analysed for the presence of peptide natural products (Ndogo Eteme et al., 2018). The Violaceae family is a well-known and rich source for small circular peptides called cyclotides (Burman et al., 2015; Park et al., 2017). Cyclotides belong to the class of ribosomally synthesized and post-translationally modified peptides (RiPPs). They are encoded by larger precursor proteins that are processed post-translationally to yield the mature peptides (Arnison et al., 2013). Cyclotides are head-to-tail cyclized peptides of approximately 30 amino acids in size containing six conserved cysteine residues as well as an Asn or Asp residue at the native backbone ligation site (Craik et al., 1999; Saska et al., 2007). The disulfide bonds form a characteristic cyclic cystine knot (CCK) motif (Craik et al., 1999). The circular peptide backbone together with the CCK motif render cyclotides extremely stable toward acidic conditions, heat-induced and proteolytic degradation (Burman et al., 2015). These peptides have many interesting bioactive properties (Ojeda et al., 2019; Grover et al., 2021), and recently cyclotides were studied as inhibitors of the prototypical post-proline cleaving enzyme prolyl oligopeptidase (POP) (Hellinger et al., 2015a).

POP is a prototypic proline specific endopeptidase with high cytosolic expression levels in several tissues, e.g. neurons and immune cells (Mannisto and Garcia-Horsman, 2017; Nagatsu, 2017). From a molecular point of view, the enzyme has high substrate specificity for motifs with only *trans*-configured proline residues in the P1 position and only modest affinities for alanine and cysteine residues (Bär et al., 2006). Small peptide substrates, usually no greater than ∼3 kDa have access to the protease binding pocket (Fülöp et al., 1998; Hellinger et al., 2015a). POP has an important role in peptide hormone homeostasis (Lambeir, 2011; Nagatsu, 2017) and many peptide hormones e.g. Arg-vasopressin, substance P, oxytocin, bradykinin and neurotensin have been identified as substrates of POP (López et al., 2011). Further, angiotensin II conversion to angiotensin (1-7) is also POP dependent and thus, POP may be relevant in renal diseases, including hypertension and cardiovascular disorders (Kaltenecker et al., 2020; Serfozo et al., 2020). POP was also being discussed as drug target in dementia, schizophrenia and amnesia; in this regard, POP inhibitors were studied in clinical trials (López et al., 2011; López et al., 2013; Mannisto and Garcia-Horsman, 2017), without any drug being translated into clinical use, yet. More recently, POP has been investigated as target for inflammation and neurodegenerative disorders, such as multiple sclerosis (Tenorio-Laranga et al., 2013) and Parkinson’s disease (Dokleja et al., 2014; Mannisto and Garcia-Horsman, 2017). Despite many research studies the evidence for the pathological role of POP in these diseases is still unclear since disease-relevant brain signaling pathways modulated by POP have not been identified to date (Mannisto and Garcia-Horsman, 2017; Nagatsu, 2017).

One common feature of various POP substrates or inhibitors are proline containing motifs, amongst them plenty of endogenous peptide hormones (Lambeir, 2011; López et al., 2011). Many POP inhibitors and substrates are thought to have more complex interaction kinetics with allosteric-like properties (Mannisto and Garcia-Horsman, 2017). A plethora of mechanistic studies on the enzyme-inhibitor complex and its molecular interaction enabled insight to substrate gating, catalysis and protein-protein interaction of POP. The understanding of conformational dynamics between POP and its substrates or inhibitors, appears as a promising tool which may result in developing new POP therapeutics (Svarcbahs et al., 2019). On the other hand, ‘drug discovery from nature’ has been a promising strategy (Atanasov et al., 2021) for the discovery of novel enzyme inhibitors (Hellinger and Gruber, 2019).

Previous phytochemical studies identified natural products as inhibitors for POP such as small molecule entities and natural peptides. For example, aqueous extracts of plants used in traditional Chinese medicine (Tezuka et al., 1999; Tarragó et al., 2006) as well as alkaloids (e.g. californidine found in *Eschscholzia californica* Cham. or berberine found in different plant species such as *Berberis vulgaris* L.) or the flavonoids (e.g. balcalein) inhibit POP activity (Tarrago et al., 2007; Tarragó et al., 2008; Cahlíková et al., 2015). Importantly, plant peptides have been shown to inhibit POP such as the trypsin inhibitor peptide bevuTI-I isolated from *Beta vulgaris* L. (Retzl et al., 2020), or cyclotides. The cyclotides psysol 2 (*Psychotria solitudinum* Standl.) and kalata B1 (amongst others in *V. tricolor* L.) were shown to inhibit POP with a half maximal inhibitory concentration (IC_50_) of 25 µM and an IC_50_ of 5.6 µM respectively (Hellinger et al., 2015a). However, nature-derived peptides remain largely underexplored and major efforts are needed to continue discovery of novel inhibitors for well-establishedprotease drug targets, e.g. post proline endopeptidases (Drag and Salvesen, 2010; Farady and Craik, 2010; Hellinger and Gruber, 2019). 

In the present study, the active molecules of plants used in African traditional medicine were deciphered using chemical extraction, peptidomics and protease inhibition experiments. We isolated novel cyclotides from Allexis species and studied their POP protease inhibiting activity. The cyclotide content in four species was analyzed by chemical derivatization, MALDI TOF mass spectrometry and reversed phase (RP)-HPLC. Protease inhibition of the plant extracts, and a bioassay-guided fractionation approach yielded two purified peptides. These molecules were further analyzed by peptidomics for obtaining their amino acid sequence and their inhibitory potency towards human POP was quantified. A comparative analysis of inhibitor sequences provided a first glimpse of the sequence-activity relationship of cyclotide POP inhibitors. Since, POP has potential relevance as a drug target in several diseases, the present study enables future research about the discovery and design of novel plant-based POP inhibitors.

## Materials and Methods

### Chemicals and Reagents

Acetonitrile (AcN), methanol (MeOH), dichloromethane (DCM), trifluoroacetic acid (TFA), and H_2_O were purchased as HPLC grade from Carl Roth (Karlsruhe, Germany). Dithiothreitol (DTT), α-cyano-hydroxy cinnamic acid, trypsin (sequencing grade), and iodoacetamide (IAA) were purchased as Bioultra grade (Sigma-Aldrich, Vienna, Austria). Endoprotease Glu-C (GluC), trypsin and chymotrypsin (sequencing grades) were purchased from New England Biolabs (Ipswich, MA). Z-Gly-Pro-amino-coumarin was purchased from Bachem (Bubendorf, Switzerland).

### Plant Material

Samples of *Allexis cauliflora* (Oliv.) Pierre*, Allexis batangae* (Engl.) Melch, *Allexis obanensis* (Baker f.) Melch and *Allexis zygomorpha* Achound. & Onana were harvested at ‘Mont des Elephants’ (coordinates: 2° 48.26′ N, 10° 02.10′ E, Alt: 85 m), 27 km from Edea in Kribi, southern province of Cameroon, by Mr. Nana from the National Herbarium of Yaoundé (Cameroon). A sample of each species was deposited at the National Herbarium of Yaounde in Cameroon under the reference: 18374/NHC for *A. cauliflora*, 31839/NHC for *A. batangae*, 49778/NHC for *A. obanensis* and 30,421/NHC for *A. zygomorpha*.

### Extraction of Plant Peptides

The plant material was dried and stored at 25°C in a humidity-free environment. To obtain a powder for extraction, the dried plant material was crushed using a kitchen grinder. The powder was extracted with 1:1 (v/v) DCM/MeOH mixture with continuous stirring at room temperature for 24 h. The liquid extraction solution was separated from the solid material by filtration, and the liquid introduced into a separatory funnel. Then, 0.5 vol. of double distilled H_2_O (dd H_2_O) was added for the liquid-liquid phase extraction to separate the aqueous phase containing the peptides from the organic phase. The extract was then batch processed by solid phase extraction (SPE) with octadecyl modified silica gel (Zeoprep 60 Å, irregular C_18_ material 40–63 μm from Zeochem, Uetikon, Switzerland) similar as described earlier (Hellinger et al., 2015b; Tam et al., 2015). The C_18_ material was preconditioned (activation and cleaning) with one column volume of MeOH and twice with buffer A (100% ddH_2_O/0.1% TFA, v/v). The crude extract solution in buffer A was loaded onto the SPE column. Subsequently, three column volumes of 20% AcN in buffer A were used to remove the non-adherent and polar plant constituents. The peptide-containing fraction was eluted with 80% AcN in buffer A. The eluate was lyophilized and will be referred to as ‘peptide enriched extract’.

### High Performance Liquid Chromatography

The peptide enriched extract was further purified via reversed phase (RP) HPLC. The mobile phase for all HPLC analysis consisted of solvent A (100% ddH_2_O/0.1% TFA, v/v) and solvent B (90% acetonitrile/10% ddH_2_O/0.1% TFA; v/v/v). The dried extract was dissolved in solvent A and fractionation of the extract and purification of cyclotides were carried out on a preparative and semi-preparative scale using a Kromasil C_18_ column (250 × 21.2 mm, 10 μm, 100 Å or 250 × 10 mm, 5 μm, 100 Å; diChrom GmbH, Germany). Elution of the analyte was monitored by UV absorbance at 214, 254, and 280 nm. The fractions were collected automatically by time or manually by peak. Analytical HPLC was performed accordingly, using a Kinetex C_18_ column (150 mm × 3.0 mm, 2.6 μm, 100 Å, Phenomenex) at a flow rate of 0.4 ml min^−1^.

### MALDI-TOF/TOF Mass Spectrometry

Peptide enriched extracts and HPLC fractions were analyzed via MALDI-TOF mass spectrometry (MS) to evaluate the samples mass signals in the mass range of 2,500–4,000 Da. The analysis was performed on a 4800-type analyzer in positive reflector mode from Sciex (Framingham, MA) or on an autoflex speed TOF/TOF MALDI-MS System from Bruker (Bremen, Germany). Prior to analysis, the samples were mixed with a saturated matrix solution of α-cyano-hydroxy cinnamic acid dissolved in AcN/ddH_2_O/TFA 50/50/0.1% (v/v/v) at a ratio of 1:6, and 0.5 μL of this mixture was transferred onto the target plate to allow air-drying in the darkness. Mass accuracy was ensured by daily calibration using Peptide Mix 4 (Laser Biolabs, Valbonne, France). For each spot 2,500–3,000 shots were recorded and accumulated to a summed spectrum similar as described in published protocols (Koehbach et al., 2013; Hellinger et al., 2015a; Hellinger et al., 2015b).

### Screening for Cysteine-Rich Peptides

Peptide enriched extracts were evaluated using established peptidomics workflows to elucidate the number of cysteines in the peptide and a cyclic backbone (Koehbach et al., 2013; Hellinger et al., 2015b; Tam et al., 2015) with few modifications. Samples were prepared in 0.1 M NH_4_HCO_3_ buffer pH 8.2. Reduction of disulfide bonds was performed with 15 mM DTT at 37°C for 45 min. The reactive sulfhydryl groups were derivatized with 75 mM IAA at room temperature for 10 min in darkness. Excess of alkylating reagent was quenched with the addition of DTT. Compared to the native precursor peptide a mass shift of +58 Da per reacted residue is observed after reduction and alkylation. For confirmation of the cyclic backbone, the S-carbamidomethylated samples were site-specifically processed with endoprotease GluC at 37°C for 16 h. Enzyme activity was quenched with addition of acetic acid 3% (v/v) final concentration. The mass shift for a single cleavage site in a cyclic peptide is +18 Da compared to the native analyte. The described mass shifts were evaluated in the recorded MS spectra with a tolerance of ±25 ppm to the native mass of the compound.

### MS/MS Fragmentation and *de Novo* Peptide Sequencing

Isolated pure peptides (50 µg) were treated with DTT and IAA as described above. The cyclic alkylated species were proteolytically digested with trypsin (0.2 µg) or endoprotease GluC (0.25 µg) at 37°C or chymotrypsin (0.2 µg) at room temperature overnight or as indicated in the figure legends. The samples were de-salted and concentrated using ZipTip® purification tips according to the manufacturer protocol (Merck-Millipore, Germany). Analysis of observed fragment peptides from site specific proteolytic cleavage experiments enabled the annotation of isobaric amino acids, for instance Ile and Leu or Gln and Lys, respectively. MS/MS fragmentation experiments used post-source decay events of identified precursors derived from cyclotide proteolysis. The obtained fragmentation spectra were used for *de novo* interpretation of the amino acid sequence. The y-ion and b-ion specific losses, such as −17 or −18 Da were considered for residue annotation. High sensitivity amino acid analysis was performed by the Australian Proteome Analysis Facility (Macquarie University, Sydney, Australia). In brief, following gas phase hydrolysis of the samples in 6 M hydrochloric acid at 110°C, the amino acids were labelled using Waters AccQTag Ultra chemistry and analyzed by UPLC. The molar absorption coefficients for alca peptides were calculated with 1865 M^−1^ cm^−1^ using the ExPASy ProtParam tool (Swiss Institute of Bioinformatics) (Wilkins et al., 1999). The mass spectrometry MALDI-MS and MS/MS data have been deposited to the ProteomeXchange Consortium via the PRIDE partner repository with the dataset identifier PXD026664.

### Protease Inhibition Assay

The POP inhibition assays were carried out similarly as recently described (Hellinger et al., 2015a; Retzl et al., 2020) using a bacterial expressed POP enzyme. Human POP cDNA (accession NM:002726.4) was inserted into a pGEX-6P1 expression vector using the EcoRI and XhoI sites (Genescript, Leiden, Netherlands) and expressed in *Escherichia coli* (BL21 D3 strain). Protein expression was induced by addition of 0.3–0.5 mM isopropyl-β-d-thiogalactopyranoside (ProteinArk, Rotterdam, Netherlands) at an optical density (OD600) between 0.6 and 0.8 to the bacterial culture and incubated at 25°C under constant shaking. The protein was harvested at an OD600 between 1.4 and 1.5. POP was purified via GST-affinity capture beads. After protein binding three wash steps were performed with incubation buffer and PreScission protease was used for site-specific cleavage releasing the untagged protein from the resin. The protein concentration was determined with bicinchoninic acid assay (ThermoFisher). The protein was stored at −80°C in storage buffer 25 mM Tris-HCl, pH 7.9, 250 mM sodium chloride, 20% (v/v) glycerol, 1 mM DTT. For enzyme inhibition assays human POP (50 ng/well) was incubated in assay buffer (20 mM Hepes pH 7.2, 150 mM NaCl, 1 mM EDTA, 0.5 mM DTT) together with substrate Z-Gly-Pro-amino-coumarin (final concentration 45 µM) at 37°C for 60 min. The assay conditions are similar as described in studies using POP isolated from porcine brain (Venäläinen et al., 2002) or human plasma samples (Bracke et al., 2019). *K*_m_ and V_max_ of the bacterial enzyme have been validated. All experiments were performed in 96-microtiter black bottom plates. Concentration-response inhibition assays were performed with [S] = *K*_m_, which is considered a suitable condition for evaluating samples of unknown inhibition mode (e.g. competitive, noncompetitive or uncompetitive reversible inhibition). The protease activity was quantified as fluorescence from the proteolytically cleaved amino-coumarin fluorophore with excitation 380 nm and emission 420 nm on a Biotek Synergy H4 plate reader. The fluorogenic substrate provide elevated fluorescence by the delocalization of electrons after the amide bond cleavage and release of the 7 amino coumarin moiety. All samples for protease inhibition assays were prepared in ddH_2_O. Concentration-dependent inhibition studies were performed with the peptide enriched extracts (2–1,000 μg/ml) or isolated peptides (0.1–60 µM). For bioassay-guided isolation HPLC fractions were assayed for POP inhibition with three concentrations (4, 20 and 100 μg/ml). Kinetic measurements were carried out to obtain initial velocities in the presence of sample (v_i_) or enzyme alone (v_o_) (Arnison et al., 2013). The percent remaining POP activity was calculated as I = (v_i_/v_o_)*100. A substrate background was used for correction of all measurements. KYP-2047, a specific POP inhibitor, was used as positive control in the assays. For the graphical illustration, the inhibition data of extracts or isolated peptides were normalized to the maximum response. The inhibition of HPLC fractions in the bioassay-guided isolation experiment was normalized to the full enzyme response (100% is the enzyme activity). All data are represented as mean of three to six independent experiments ± standard deviation for the fraction or the purified peptide enriched extract, respectively. Four parameter non-linear regression curve fits were obtained using GraphPad Prism v5.0 fitting algorithms with equation Y=Bottom + (Top-Bottom)/(1 + 10^((LogIC50-X)*Hill Slope)) in which X is the log dose and Y the measured response.

### Homology Analysis, Sequence Alignments, Sequence Logos and Cyclotide Cartoons

Sequence logos were prepared using the WebLogo tool (Crooks et al., 2004). Cyclotide sequences were obtained from cybase (Hashempour et al., 2013). Representative Violaceae cyclotide sequences, excluding mixed type, partial as well as linear sequences, were used for the sequence logo. The cyclotide sequences were sorted into *Moebius* type (87 sequences) and bracelet type (189 sequences) cyclotides, based on the presence or absence of a *cis-*Pro residue in loop 5. The structural models were generated with PyMol using the published PDB data of kalata B1 (1NB1) (Rosengren et al., 2003) and cycloviolacin O2 (2 KNM) (Göransson et al., 2009). To identify cyclotides with high similarity to alca 1 and alca 2, pairwise alignments of the novel peptides to reported cyclotides were scored (identical character score 1, no gap penalties) as a measure for sequence homology. The scoring used all cyclic cyclotide sequences downloaded in FASTA-format from cybase. The pairwise alignment was performed using the Biopython pairwise2 module (Cock et al., 2009). For precursor gene analysis all available precursor genes of Violaceae species were retrieved from cybase (date of access: 9th of July 2020). The sequences of all regions were aligned with Clustal Omega (standard parameters) using the European Bioinformatics Institute search and sequence analysis tools application programming interface (Wang et al., 2008) and visualized with frequency logos. The length of all regions were analyzed with custom Python scripts.

## Results

### Identification of Cyclic Cystine-Rich Peptides in *Allexis* Species

**FIGURE 1:**
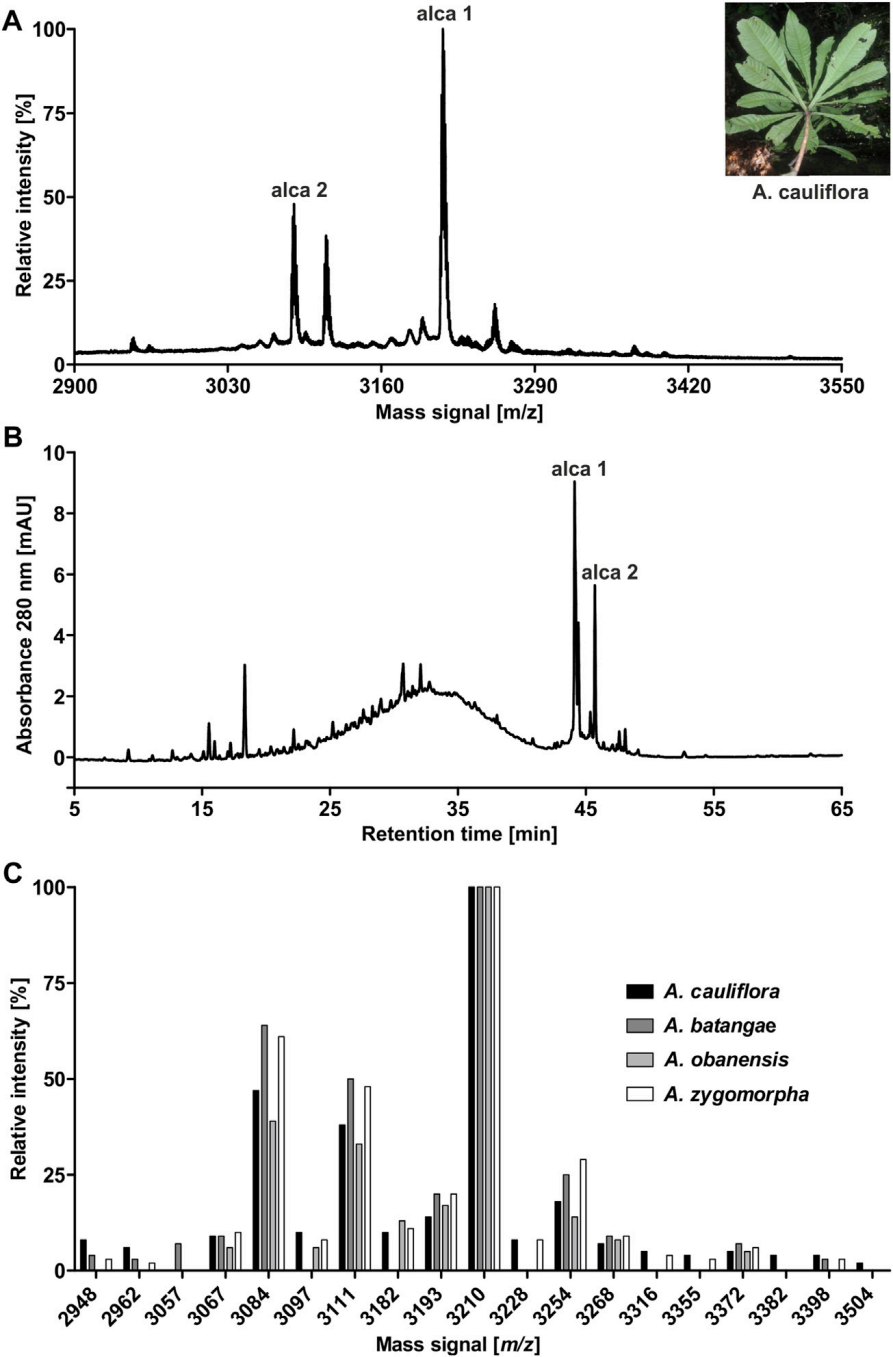
Identification of peptides in extracts of *A.* *cauliflora*. MALDI-TOF-MS analysis was performed to evaluate mass signals of *A. cauliflora* peptides and other mid-weight molecules in the range of 2,900–3,550 Da. The two major signals were retrospectively denoted as alca 1 (*m/z* 3,211.4) and alca 2 (*m/z* 3,084.2) in the spectrum. A representative photograph of *A. cauliflora* is shown in the insert (property of David Kenfack under CC BY-NC 4.0 licence, http://korupplants.myspecies.info/) **(A)**. The analytical HPLC chromatogram of the *A. cauliflora* extract at 280 nm absorbance is shown in **(B)**. Mass signals in the interesting range of 2,900–3,550 Da of four Allexis species, *A. cauliflora, A. batangae, A. obanensis* and *A. zygomorpha,* were compared as relative intensities of observed mass signals. For the comparative plot each mass signal in the spectra was independently normalized to the highest signal **(C)**.

**FIGURE 2:**
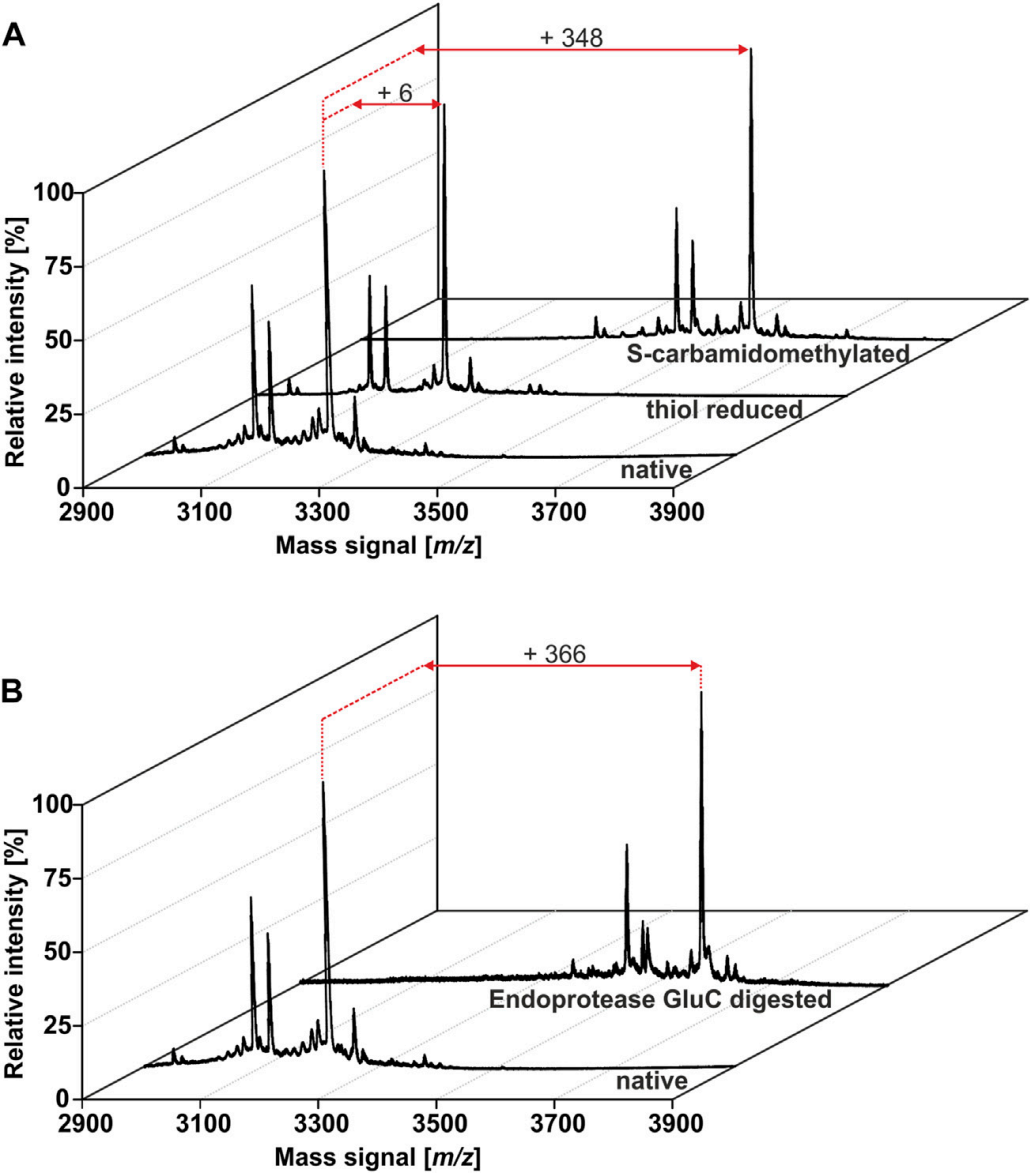
Identification of cyclic cystine-rich peptides in *A.* ***cauliflora.*** A cystine identification workflow was applied to evaluate the number of cysteine moieties and the presence of a single glutamic acid in the peptides. MALDI-TOF-MS analysis of native, DTT and IAA treated samples enabled the monitoring of characteristic mass shifts of +1 Da and +58 Da per reduced and acetamidated cysteine, respectively. The peptide analysis revealed the presence of in total 18 peptides with six cysteines in *A. cauliflora* **(A)**. Furthermore, a site-specific proteolytic cleavage using endopeptidase GluC obtained an additional mass shift of +18 Da (total of +366 Da) compared to the native mass for all 18 peptides. This result indicates a single conserved Glu in these peptides and due to the absence of other proteolytic fragments the experiment pinpoint on peptides with a cyclic backbone, e.g. cyclotides **(B)**.

The African flora, especially plants of Cameroon, are a rich source for identification of novel bioactive molecules. To date, Violaceae from Cameroon have not been investigated for the occurrence of RiPPs. Hence, the existence of peptides from four Allexis species was explored by applying well-established workflows for peptide natural product drug discovery (Koehbach et al., 2013; Hashempour et al., 2013). All plant material was collected in the field at Mont des Elephants in Kribi in the southern province of Cameroon. The species *A. batangae*, *A. cauliflora*, *A. obanensis* and *A. zygomorpha* were identified with the help of a local botanist. The preparation of the peptide enriched extracts by solvent extraction and subsequent C_18_ solid phase extraction resulted in the enrichment of amphiphilic plant compounds such as peptides. From the starting plant material used for extraction, an average yield of approx. 0.3–0.5% of peptide enriched extract was achieved. MALDI-TOF MS analysis of the peptide enriched extract samples revealed several signals in the mass range of 2,900–3,550 Da (Figure 1A; Table 1; Supplementary Figures 1A,C,E; Supplementary Tables 1–3) and several peaks with a retention time between 40 and 50 min using analytical RP-HPLC chromatograms were observed (Figure 1B; Supplementary. Figures 1B,D,F); both analysis depicted characteristic features of cyclotides (Koehbach et al., 2013). Interestingly, all four Allexis species had similar mass signals and HPLC peak patterns, which was confirmed in a comparative analysis (Figure 1C). At least 19 different peptides were identified by MS in these species, whereof at least eight were present in all four species including, the three major peptides with *m/z* 3210, 3084 and 3111 as determined by their relative abundance. To further characterize these peptides, a chemical derivatization strategy was applied to determine the number of cysteines. By reduction of disulfide bonds with DTT to the reduced sulfhydryl groups, mass shifts of +6 Da compared to the native sample were observed in the mass spectra of all four species. Furthermore, the reduced sample was derivatized with IAA, which reacts with free sulfhydryl groups to *S*-acetamide resulting in a mass shift of +58 Da per cysteine residue compared to the native mass. The mass shifts of +348 Da for analytes in the Allexis samples indicated six cysteine residues in these peptides (Figure 2A; Table 1; Supplementary Figure 2; Supplementary Tables 1–3). As different plant peptide families are known to comprise cystine motifs containing six residues (Tam et al., 2015) the presence of cyclotides was confirmed by a site-specific proteolytic cleavage with endoprotease GluC. Following reduction, alkylation and digestion of the obtained mass signals yielded +366 Da compared to the native sample, which implies a single cleavage site (glutamic acid) present in the Allexis peptides (Figure 2B; Table 1; Supplementary Figure 2; Supplementary Tables 1–3); this is characteristic for the ‘ring opening’ in cyclotides (Koehbach et al., 2013). With the help of this peptidomics identification workflow, at least 18 peptides in *A. cauliflora*, 12 in *A. batangae*, 10 in *A. obanensis* and 16 in *A. zygomorpha* were found that contained a circular backbone and six cysteine residues (Table 1; Supplementary Tables 1–3).

**TABLE 1:** Peptidomic analysis of *A. cauliflora* peptide enriched extracts.

Native mass signal (*m/z*)^a^	Mass signal of cysteine reduced sample (*m/z*)^b^	Mass signal of cysteine carbamidomethylated sample (*m/z*)^c^	Mass signal of EndoGluc processed (*m/z*)^d^
**2,948.1**	2,954.1	3,296.3	3,314.3
**2,962.1**	2,968.1	3,310.2	3,328.3
**3,067.2**	3,073.2	3,415.3	3,433.4
**3,084.2**	3,090.2	3,432.3	3,450.4
**3,097.2**	3,103.2	3,445.3	3,463.4
**3,111.2**	3,117.3	3,459.4	3,477.4
**3,182.4**	3,188.4	3,530.6	3,548.6
**3,193.3**	3,199.3	3,541.5	3,559.6
**3,210.3**	3,216.3	3,558.4	3,576.5
**3,228.0**	3,234.0	3,576.2	3,594.2
**3,254.2**	3,260.3	3,602.4	3,620.5
**3,268.2**	3,274.2	3,616.5	3,634.4
**3,316.3**	3,322.3	3,664.4	3,682.5
**3,355.3**	3,361.3	3,703.5	3,721.5
**3,372.3**	3,378.3	3,720.5	3,738.6
**3,382.3**	3,388.2	3,730.4	3,748.5
**3,398.3**	3,404.3	3,746.5	3,764.5
**3,504.4**	3,510.4	3,852.5	3,870.5

a monoisotopic mass signals [M + H]^+^ are shown.

b Full cysteine reduction with DTT obtains a mass shift of +1.0079 Da per residue.

c Carbamidomethylation with IAA leads to a total mass shift of +348.1757 Da for 6 cysteine residues.

d Proteolytic cleavage of endoprotease GluC provides a mass shift of +18 Da corresponding to a conversion of a cyclic to a linear fragment peptide.

### Effects of Peptide Enriched Extracts of Allexis Species on the Activity of Human Prolyl Oligopeptidase

**FIGURE 3:**
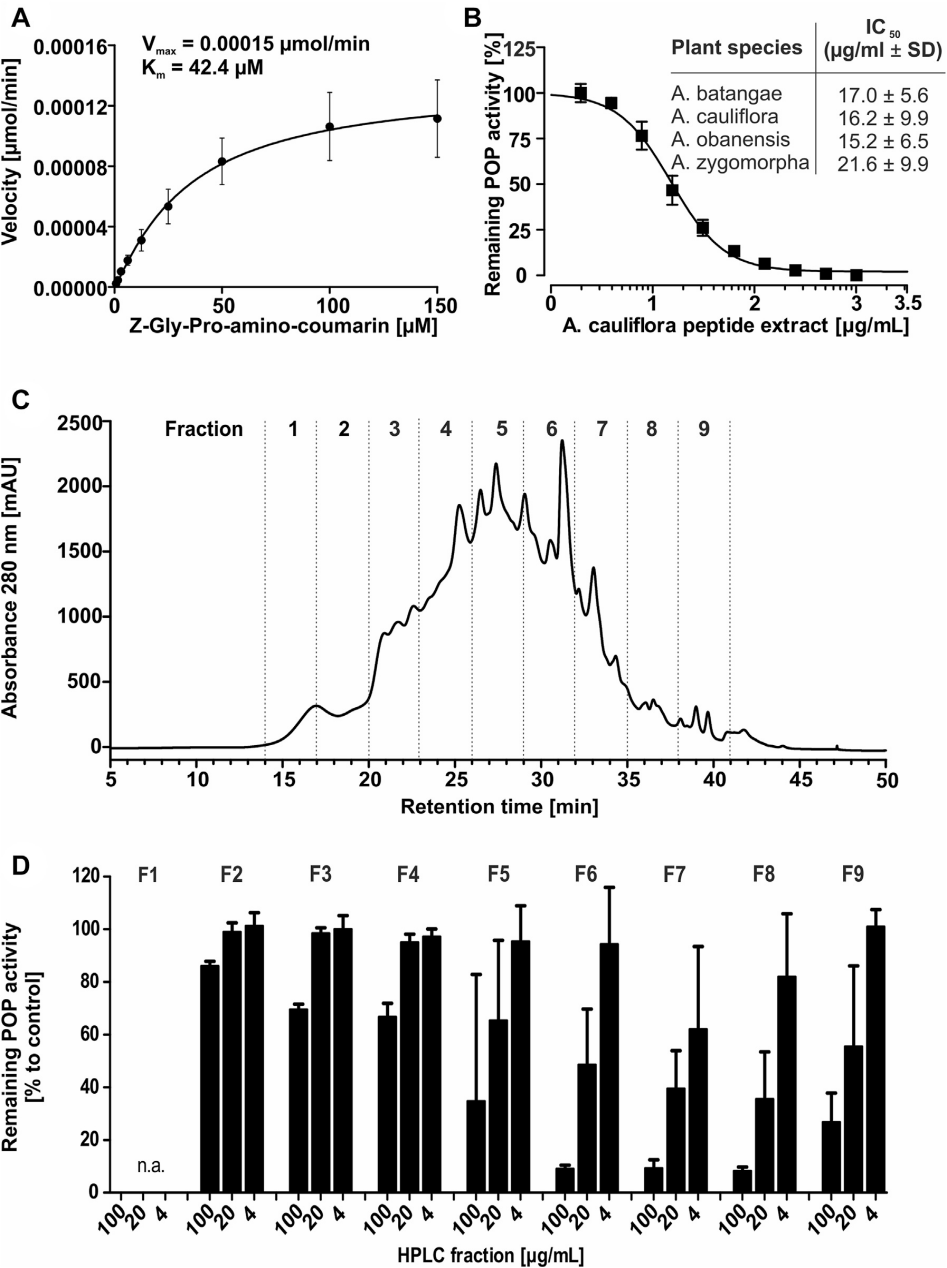
Bioassay-guided isolation of prolyl oligopeptidase inhibitors. A Michaelis-Menten kinetics were measured to ensure the quality of the recombinant enzyme; this yielded *K*_m_ (42.4 µM) and V_max_ (0.00015 µmol AMC min) using the optimized POP enzyme inhibition assay conditions **(A).** The inhibition activity of peptide enriched plant extracts derived from four specimen of the Allexis tribe towards human POP was evaluated in a dose concentration-response study applying 2–1,000 μg/ml. For *A. cauliflora* the concentration-response curve is shown. The table lists obtained IC_50_ values for the four *A. cauliflora* (IC_50_ 16.2 ± 9.9 μg/ml), *A. batangae* (IC_50_ 17.0 ± 5.6 μg/ml)*, A. obanensis* (IC_50_ 15.2 ± 6.5) and *A. zygomorpha* (IC_50_ 21.6 ± 9.9 μg/ml). All data are shown as sextuplicate experiment including ±standard deviation **(B)**. A bioassay-guided fractionation of the most representative peptide enriched extract of *A. cauliflora* was applied to identify and isolate peptide inhibitors of POP. A chromatogram with the recorded A_280_ signal of a preparative RP-HPLC experiment shows the fractionation. The prepared fractions (denoted as F1, −2, −3 to F9.) are separated with dotted lines **(C)**. Fraction material for sample F1 yielded ≤2 mg and was not analysed. The fractions were evaluated with the concentrations of 4, 20, and 100 μg/ml. The highest inhibition was detected for F7<F6<F8∼F9<F5, whereas other fractions showed little or no inhibition (F2-4). KYP-2047 was used as control inhibitor (data not shown). The inhibition activity is expressed relative to the non-inhibited control (100% enzyme activity). All data are shown as quadruplicate experiment including ±standard deviation **(D)**.

Previous studies confirmed that cyclotides are protease inhibitors, including inhibition of POP (Hellinger et al., 2015a). Accordingly, peptide extracts of all four Allexis species were characterized for their effect toward the proteolytic activity of human POP. Functional human POP protein was expressed in *Escherichia coli*, purified by affinity chromatography and the full-length untagged protein was obtained after site-specific cleavage (Supplementary Figure 3A). The assay conditions were optimized to ensure maximum enzymatic activity (Supplementary Figures 3B–G). The identified parameters for buffer system, pH value, effects organic solvents (from substrate stock solution) (Tarragó et al., 2006) as well as DTT (Venäläinen et al., 2002; Bracke et al., 2019) and NaCl concentration (Szeltner et al., 2002) on POP enzyme activity were similar as reported in previous studies. For example, POP activity is higher with DTT in the buffer and the enzyme activity is dependent on salt and the pH (Venäläinen et al., 2002; Szeltner et al., 2002). Based on the performed optimization the highest enzymatic activity was obtained with the assay buffer: 20 mM Hepes pH 7.2, 150 mM NaCl, 1 mM EDTA, 0.5 mM DTT. A Michaelis-Menten plot was generated and *K*_m_ of 42.4 µM and V_max_ of 0.00015 μmol/min were obtained (Figure 3A), which are in agreement with published data. The performed protease inhibition assay is based on the system described by Toide et al. (1995) and updated protocols were described previously (Hellinger et al., 2015a; Retzl et al., 2020). Using a 96-well plate assay, enzyme activity was determined in the presence of different concentrations of plant extracts. The peptide containing samples revealed a concentration-dependent inhibition of POP activity in the tested concentration range of 1.95–1,000 μg/ml. The highest activities were detected with IC_50_ 15.2 ± 6.5 μg/ml and 16.2 ± 9.9 μg/ml for *A. obanensis* and *A. cauliflora*, respectively, whereas *A. batangae* and *A. zygomorpha* extracts resulted in IC_50_ values of 17.0 ± 5.6 and 21.6 ± 9.9 μg/ml, respectively (Figure 3B; Supplementary Figure 4). Since all four Allexis extracts contained a similar peptide profile and activity to inhibit POP, we used *A. cauliflora* as representative species for bioactivity-guided isolation and purification of cyclotides.

### Bioactivity-Guided Fractionation of *A. cauliflora* Peptide Extracts

**FIGURE 4:**
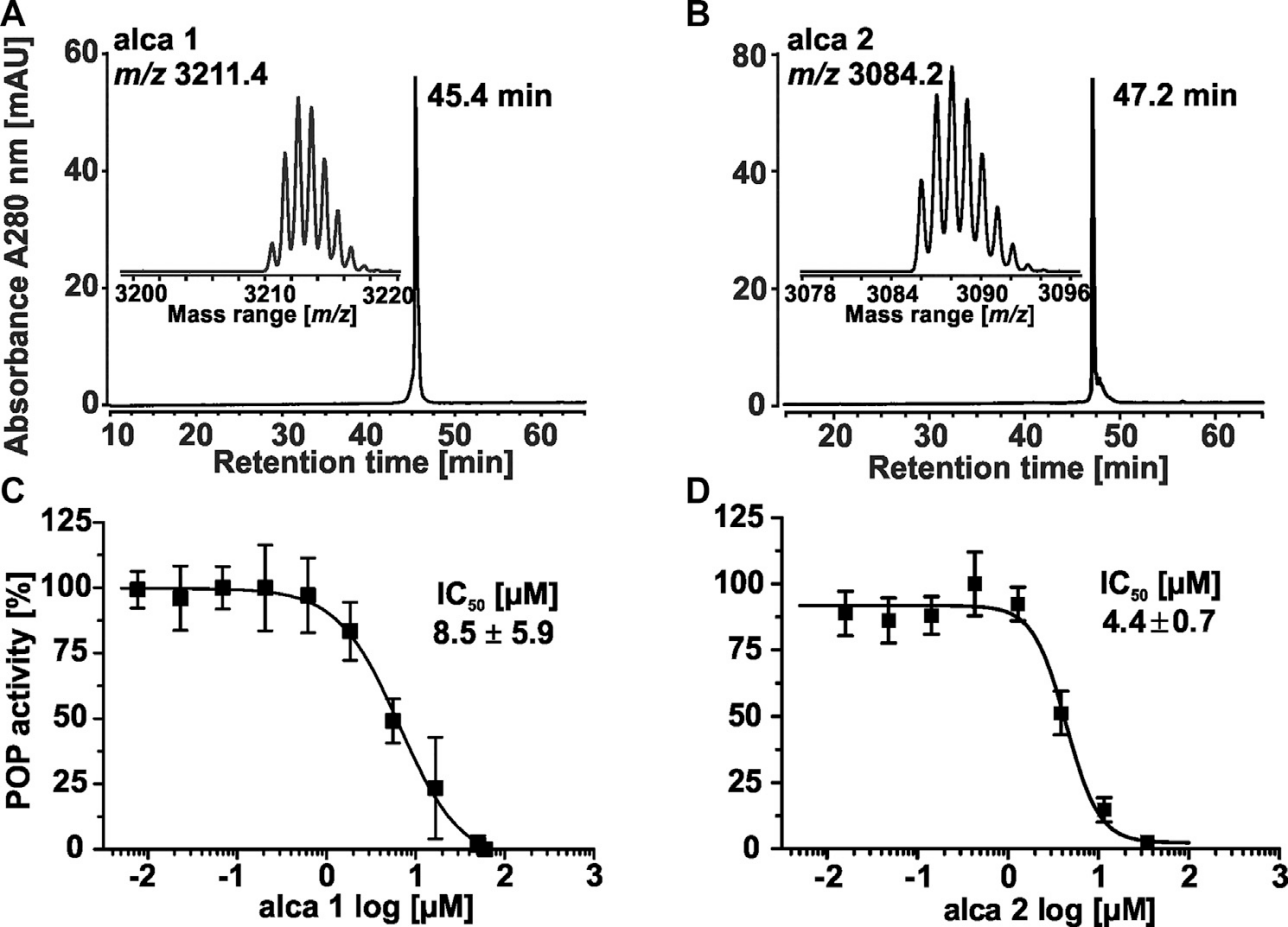
Isolation of representative alca peptides for concentration dependent inhibition of POP activity. Two peptides (denoted as alca) were obtained in the milligram scale by repeated preparative and semipreparative HPLC fractionation. The major peptides in bioactive fraction 6 (alca 1) and in −7 (alca 2) were purified with RP-HPLC. The A_280_ trace of a chromatogram allowed purity determination for alca 1 with ≥99%. The mass trace of the isolated material is shown in the insert. The native monoisotopic detected mass is m/z 3,211.4 **(A)**. The corresponding A_280_ trace for alca 2 obtained a purity of ≥82%, which was considered for calculation of peptide concentration **(B)**. A dose response study was conducted to evaluate the inhibition activity (IC_50_) of isolated peptides. POP proteolytic activity inhibition assays obtained IC_50_ 8.5 ± 5.9 µM with a Hill slope of −1.2 ± 0.1 for alca 1 **(C)** and IC_50_ 4.4 ± 0.7 µM with a Hill slope of −2.8 ± 0.9 for alca 2 **(D)**. All experiments were performed in three or five biological experiments, respectively and the data are shown as mean ± standard deviation. The inhibition was quantified to full enzyme activity and normalized to the highest measured data value for the shown plot of dose response data.

To isolate the most active peptides from *A. cauliflora* a bioactivity-guided fractionation approach for POP inhibition was used. About 2 g of peptide enriched extract was used for multiple HPLC experiments generating nine fractions with 3 min collection intervals (Figure 3C). The combined fractions were evaluated with analytical HPLC as well as by MALDI-MS analysis (Supplementary Figures 5–6). Fraction 1 did not yield sufficient material to allow further analysis. Therefore, eight HPLC fractions were evaluated for POP inhibition in a bioassay-guided analysis using the test concentrations of 4, 20 and 100 μg/ml. Fractions 2-4, with only traces of cyclotides detected by analytical HPLC, had only minor protease inhibition *i.e.* even at the highest concentration tested (100 μg/ml) there is >60% of protease activity remaining (Figure 3D). Fractions F5 and F9 exhibited moderate POP inhibition whereas fractions F6 to F8 were the most active samples, which exhibited a concentration-dependent POP inhibition with >85% POP inhibition at 100 μg/ml (Figure 3D). Considering the analytical data (RP-HPLC and MALDI-MS) of these fractions it became clear that fractions F6 and F7 contained one major peptide each (Supplementary Figures 6A–D), which could readily be purified and characterized. Therefore, we used fractions 6 and 7 to further isolate two bioactive peptides, denoted as alca 1 and alca 2, with a yield of 16.5 and 4.7 mg and a purity of >95 and 82%, respectively (Figures 4A,B). Alca 1 was characterized with *m/z* 3,211.4 [M + H]^+^ and a retention time of 45.4 min and alca 2 with *m/z* 3,084.2 [M + H]^+^ and 47.2 min, respectively. The purified peptides were utilized for measuring concentration-dependent inhibition of human POP, resulting in IC_50_ 8.5 ± 5.9 µM (alca 1) and IC_50_ 4.4 ± 0.7 µM (alca 2) (Figures 4C,D). Hill slopes of the sigmoidal inhibition curves were calculated as −1.2 and −2.8 for alca 1 and alca 2, respectively.

### Enzymatic Digestion and *de Novo* Sequencing of Alca Peptides

**FIGURE 5:**
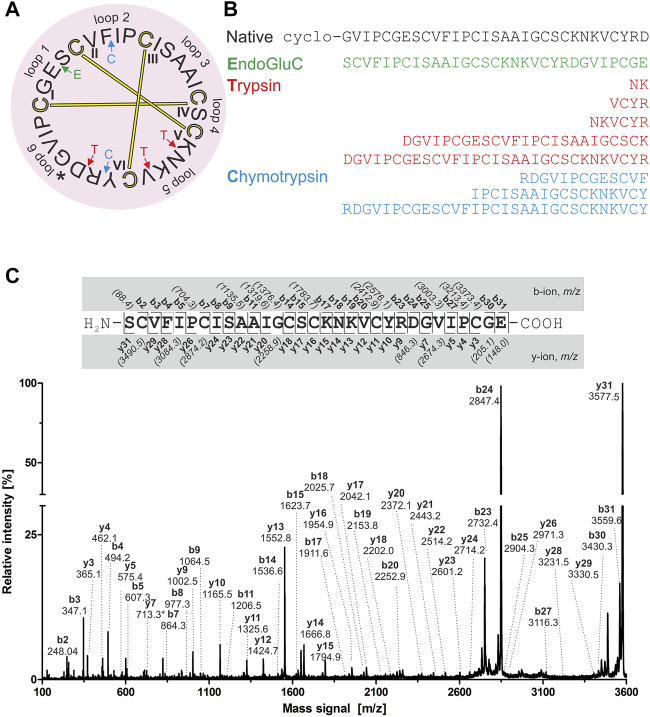
Amino acid sequence analysis of alca 1. Isolated alca 1 peptide with native *m/z* 3,211.4 was prepared for mass spectrometric experiments with collision induced fragmentation. The determined amino acid sequence is shown in a circular plot. The observed cleavage sites for endoprotease GluC (green), trypsin (red) and chymotrypsin (blue) are indicated with arrows. The cystine knot configuration was assumed by homology to cyclotides (cysteines are numbered and connected as follows: I-IV, II-V and III-VI) and disulphides are indicated with connecting bars in yellow. The intercysteine loops are numbered with 1, 2, 3 etc., starting with the native ligation site (indicated with an asterisk) **(A)** The observed peptide fragments in MS analysis are denoted in one letter amino acid code **(B)**. The ion fragment spectra of peptide precursor *m/z* 3,577.5 derived from proteolytic cleavage by endoprotease GluC is presented including labelling of all identified fragment ions. The determined amino acid sequence is depicted on the top from N-to the C-terminus. The identified y- and b-ions are labelled (*e.g.* y_1_, y_2_, etc.) and missing signals are italic numbers in brackets **(C)**.

**FIGURE 6:**
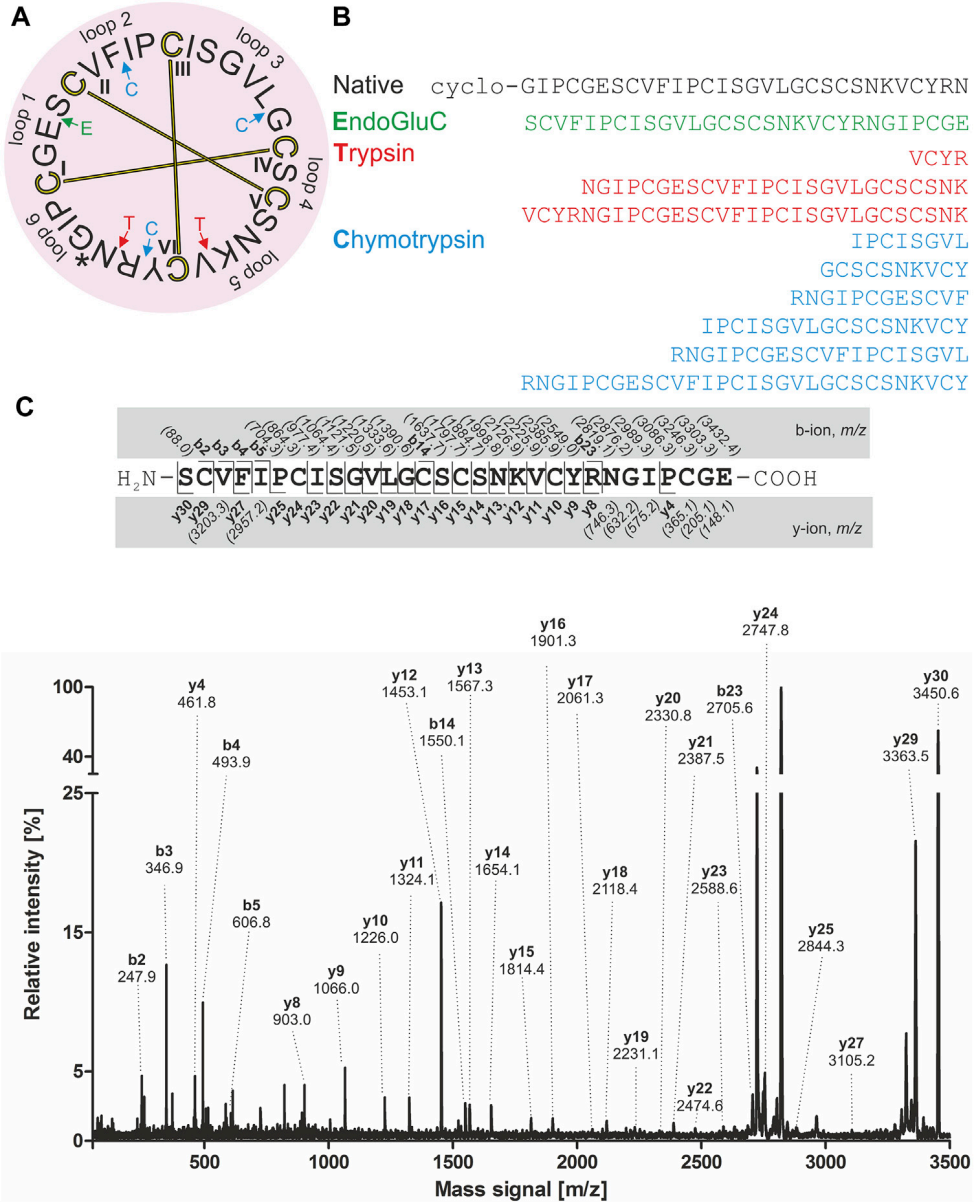
Amino acid sequence analysis of alca 2. Isolated alca 2 peptide with native *m/z* 3,084.2 was prepared for mass spectrometric experiments with collision induced fragmentation. The determined amino acid sequence is shown in a circular plot. The observed cleavage sites for endoprotease GluC (green), trypsin (red) and chymotrypsin (blue) are indicated with arrows. The cystine knot configuration was assumed by homology for cyclotides (cysteine are numbered and connected as follows: I-IV, II-IV and III-VI) and disulphides are indicated with connecting bars in yellow. The intercysteine loops are numbered with 1, 2, 3 etc., starting after the native ligation site (indicated with an asterisk) **(A).** The observed peptide fragments in MS analysis were denoted in one letter amino acid code **(B)**. The ion fragment spectra of peptide precursor *m/z* 3,450.6 derived from proteolytic cleavage by endopeptidase GluC is shown with labelling of the corresponding ions. The determined amino acid sequence is shown on the top from N-to the C-terminus. The identified y- and b-ions series are labelled (*e.g.* y_1_, y_2_, etc.) and missing signals are italic numbers in brackets. The fragmentation spectrum was assigned with all annotated ion signals **(C)**.

**FIGURE 7:**
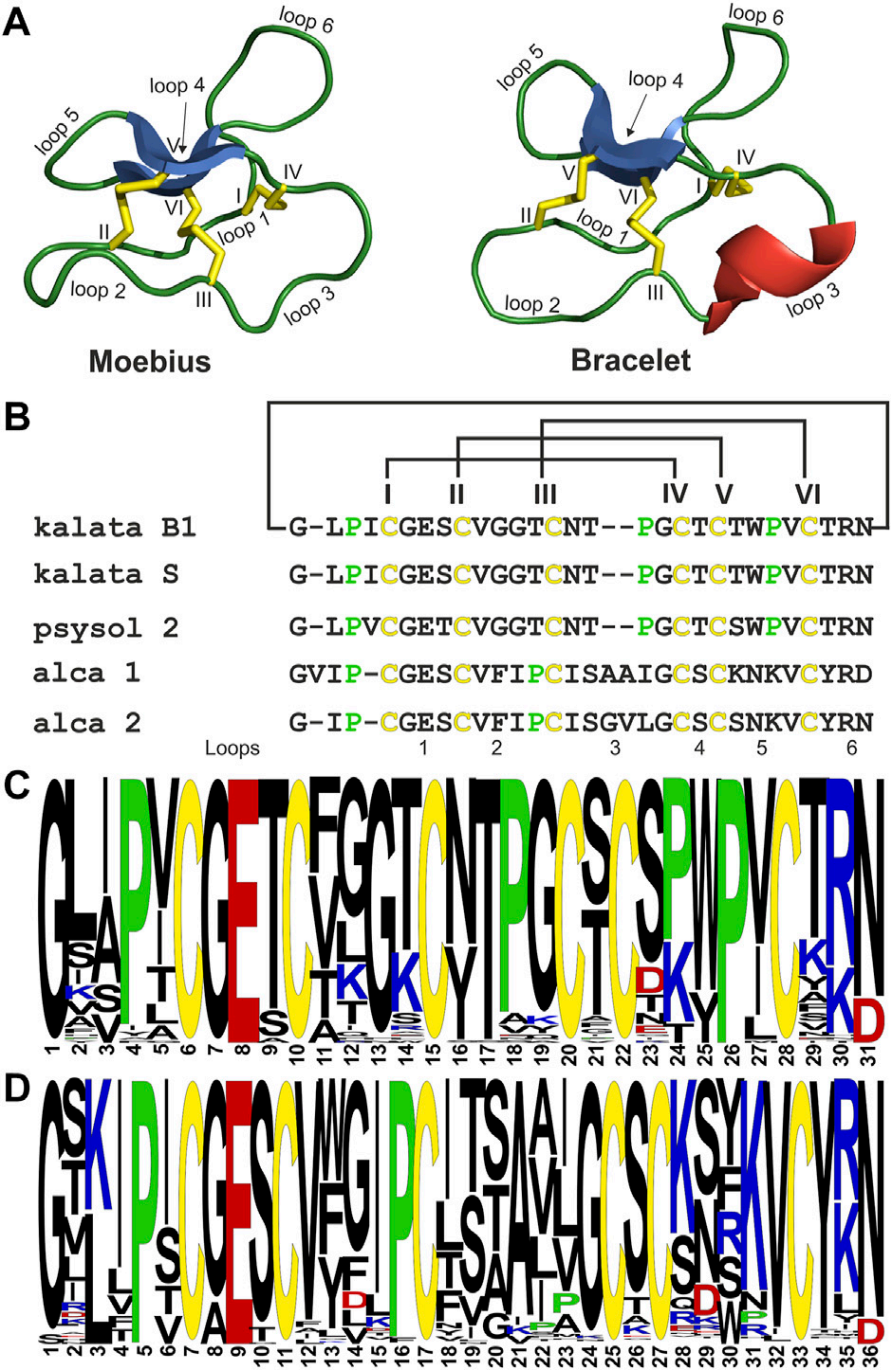
Sequence comparison of POP inhibiting cyclotides. Structural models of archetypical cyclotides kalata B1 (*Moebius* type) and cycloviolacin O2 (bracelet type) are illustrated with disulfide bonds in yellow, β-sheets in blue and α-helixes in red. Loops are labelled from 1-6 and cysteines are labelled in roman letters from I to VI **(A)**. A sequence alignment of the POP inhibitory cyclotides kalata B1, psysol 2, alca 1 and alca 2 as well as kalata S is shown. The alignment starts with the first amino acids of the mature domain. The conserved cysteines are indicated in yellow and proline residues in green. The cyclic backbone as well as the cystine knot are indicated with connecting lines. The cysteines are labelled in roman letters from I to VI **(B)**. Frequency plots of amino acid sequences of *Moebius* type **(C)** and bracelet type **(D)** cyclotides from the Violaceae family are shown. A total of 87 *Moebius* type and 189 bracelet type representative Violaceae cyclotide sequences were included for preparing the sequence logos. The conserved cysteine residues are presented in yellow, acidic in red, basic residues in blue and prolines in green. The logo starts with the first amino acid of the mature (‘core’) domain within the cyclotide precursor gene.

For *de novo* sequencing of alca 1 and alca 2, the *S-*carbamidomethylated peptides were incubated with endopeptidases to provide cleavage products for MS collision induced dissociation experiments. The analysis of the obtained proteolytic fragments using GluC, trypsin and chymotrypsin digestion has been summarized in Table 2. GluC proteolytic experiment of the peptides resulted in the fragments with *m/z* 3,577.5 for alca 1 (Figures 5A,B) and *m/z* 3,450.6 for alca 2 (Figures 6A,B). A single fragment with an increase of 18 Da of the detected mass signals results from one glutamic acid residue in the cyclic peptide backbone. Tryptic digestion of alca 1 resulted in three fragments with *m/z* 597.3, 839.5, 2,756.3 as well as *m/z* 3,577.5 for the partial cleavage product (Supplementary Figure 7A,B). Two tryptic fragments with *m/z* 597.2 and 2,872.2 were assigned for alca 2 as well as the partial cleavage product *m/z* 3,450.6 (Supplementary Figure 7C). For *de novo* sequence annotation, the full-length linear precursor peptides of the GluC digestion experiment (*m/z* 3,577.5 for alca 1 and *m/z* 3,450.6 for alca 2) were used to generate MS/MS fragmentation spectra (Figure 5C and Figure 6C). For confirmation of the obtained sequence and annotation of amino acid residues with missing ion signals in the GluC fragment spectrum, the full-length linear precursor of the tryptic digest of both peptides (*m/z* 3,577.5 for alca 1 and *m/z* 3,450.6 for alca 2) were analyzed with MS/MS fragmentation (Supplementary Figures 8, 9). The combination of sequence information of both proteolysis experiments allowed unambiguous assignment of the peptide sequences. For instance, for alca 1 full sequence coverage was achieved with at least one observed signal of the y-, or b-ion series for each of the residues (Figure 5). For alca 2, all residues were annotated based on corresponding ion fragmentation series, except one cysteine residue at position 4 (Figure 6). However, the corresponding dipeptide mass was observed in the fragmentation pattern. Isobaric Leu and Ile residues were examined by an analysis of chymotryptic fragments, which cleaves C-terminal after Leu but is sterically hindered by Ile (Supplementary Figures 7B,D). Based on chymotrypsin digest analysis, high sensitivity amino acid analysis and homology to known cyclotides (Wang et al., 2008), Ile residues at position 3, 12, 15, and 19 were assigned in alca 1. In alca 2 one Leu residue at position 18 was identified and three Ile residues at positions 2, 11, and 14 (Supplementary Tables 4, 5). Subsequently, the full-length amino acid sequences for the peptide alca 1 is cyclo-GVIPCGESCVFIPCISAAIGCSCKNKVCYRD and cyclo-GIPCGESCVFIPCISGVLGCSCSNKVCYRN for alca 2. Analysis of Violaceae precursor proteins exhibited family specific and conserved structures consisting of an endoplasmic reticulum signal (ER-signal), a pro-domain, a N-terminal repeat (NTR), a mature core domain and a C-terminal tail (C-tail). The N-terminal repeat and the mature domain can be repeated up to three times (Supplementary Figure 10). The very similar precursor topology within Violaceae is in agreement with the observed similar peptide expression pattern in the four Allexis species. Sequence comparison of the novel alca peptides with published cyclotide sequences show high similarity to other Violaceae cyclotides and a representative alignment of alca 1 and alca 2 to five similar cyclotides is shown in Supplementary Data Sheet 1. For instance, cycloviolin A and Hyfl-I share highest similarity to alca 1 and alca 2, respectively. For both, the 31-mer alca 1 and the 30-mer alca 2 a *cis*-Pro bond in intercysteine loop 5 is absent which is described for the bracelet type subfamily of cyclotides (Craik et al., 1999). Homology sequence alignment analysis of the new alca peptides with reported POP inhibitors of the *Moebius* type (Figure 7A) suggests that the alca peptides contain a putative CCK motif. This structural feature is shared amongst all cyclotides, irrespective of their subfamily; *Moebius* and bracelet cyclotides entities have structural differences, e.g. a short α-helical segment located in loop 3 of bracelet cyclotides, the CCK motif is the major structural feature determining the cyclotide fold (Figure 7B).

**TABLE 2:** Endoprotease GluC, tryptic and chymotryptic fragments of alca 1 and alca 2

Alca 1			Alca 2
**Endoprotease GluC digest**			
** [*m/z*]^a^**	**sequence** **^b^**	***[m/z]*** ^**a**^	**sequence** **^b^**
**3,577.5**	SCVFIPCISAAIGCSCKNKVCYRDGVIPCGE	**3450.3**	SCVFIPCISGVLGCSCSNKVCYRNGIPCGE
**Trypsin digest**			
**[*m/z*]^a^**	**sequence** **^b^**	***[m/z]*** ^**a**^	**sequence** **^b^**
**261.7**	*NK* ^c^		
**597.3**	VCYR	**597.2**	VCYR
**839.5**	NKVCYR	**2,872.2**	NGIPCGESCVFIPCISGVLGCSCSNK
**2,756.3**	DGVIPCGESCVFIPCISAAIGCSCK	**3,450.3**	VCYRNGIPCGESCVFIPCISGVLGCSCSNK
**3,577.9**	DGVIPCGESCVFIPCISAAIGCSCKNKVCYR		
**Chymotrypsin digest**			
**[*m/z*]^a^**	**sequence** **^b^**	***[m/z]*** ^**a**^	**sequence** **^b^**
**1,494.8**	RDGVIPCGESCVF	**858.5**	IPCISGVL
**2,101.0**	IPCISAAIGCSCKNKVCY	**1,234.5**	GCSCSNKVCY
**3,577.5**	RDGVIPCGESCVFIPCISGVIGCSCSNKVCY	**1,395.6**	RNGIPCGESCVF
		**2,074.8**	IPCISGVLGCSCSNKVCY
		**2,234.9**	RNGIPCGESCVFIPCISGVL
		**3,450.3**	RNGIPCGESCVFIPCISGVLGCSCSNKVCY

a monoisotopic [M + H]^+^ mass signals of obtained fragments are shown.

b sequence of the observed fragments after trypsin/chymotrypsin digest, annotated from N- to C-terminal end.

c this fragment was not observed in the mass spectrum.

## Discussion

Plants are valuable starting points for natural product drug discovery and development (Dias et al., 2012; Harvey et al., 2015), and the current spectrum of reported bioactive compounds may only be a glimpse of the diversity nature’s molecular treasure-trove has to offer. In particular, remote locations such as the Cameroonian flora are largely unexplored and much work remains to be done for ethnopharmacologists and chemists to identify medicinal herbs and their active ingredients (Simbo, 2010; Ntie-Kang et al., 2013). This marriage of disciplines has been successfully utilized previously for the discovery of bioactive plant peptides in Violaceae (Hellinger et al., 2014; Attah et al., 2016). The present study shines a spotlight on four species of the tribe Allexis collected in Cameroon (*A. batangae*, *A. cauliflora*, *A. obanensis* and *A. zygomorpha*), which have been used for the identification and bioactivity characterization of nature-derived peptides. Allexis have been given little attention in medicinal research. To date, phytoanalysis on Allexis led to the identification of antimicrobial molecules, which have been obtained by organic solvent extraction (Ndogo Eteme et al., 2018). In contrast, the scope of the current study was to extract plant metabolites with medium hydrophobicity (Gruber et al., 2008; Ireland et al., 2010). The enriched peptidic plant constituents expectedly were of the RiPPs class, which includes orbitides, cyclotides, defensins, knottins, thionins, heveins, snakins and other plant families (Arnison et al., 2013; Das et al., 2020). Consequently, this study used established peptidomic protocols to conduct chemical analysis and characterization of isotypic peptide libraries of the four Allexis species (Koehbach et al., 2013). For instance, the molecules were analyzed for conserved structural elements or unique sequence features, such as a cystine-knot motif or a head-to-tail-cyclized peptide backbone. A CCK motif was identified in Allexis peptides, which is the characteristic structural element for the cyclotide family. Cystine-knots are reported for knottins, snakins*,* α-amylase or potato metalloprotease inhibitors, but these are not backbone cyclic peptides (Tam et al., 2015; Correnti et al., 2018; Hellinger and Gruber, 2019). Based on the peptidomics analysis the expression of cyclotides was conclusively confirmed in Allexis species (Craik and Malik, 2013; Koehbach and Clark, 2016; White and Craik, 2016). The overall similar peptide expression in four plant species might reflect conserved cyclotide precursor genes within Allexis and cyclotide genes with two or three mature domains. Therefore, the analysis of the encoding precursor genes (Gruber et al., 2008; Koehbach and Clark, 2016; Park et al., 2017), or the processing enzymes (Montalbán-López et al., 2021) as well as the mature peptides have been used for the characterization of plant RiPPs in previous bioanalytical and molecular analysis. Peptidomics studies combining information derived from the level of nucleic acid and mass spectrometric analysis are powerful tools to explore the diversity of violaceous cyclotides in the past (Colgrave et al., 2010; Ireland et al., 2010; Hellinger et al., 2015b; Burman et al., 2015). Indeed, this plant family is one of the prototypic cyclotide expressing plants with many positively confirmed species, e.g. *V. tricolor* (Hellinger et al., 2015b), *V. odorata* (Colgrave et al., 2010), *H. enneaspermus* (Du et al., 2020) or *Rinorea bengalensis* (Wall.) Gagnep. in Humbert (Niyomploy et al., 2018), only to mention a few examples. In addition, cyclotides from Violaceae have been investigated in various biological assays and several bioactivities have been reported to date, such as antimicrobial (Tam et al., 2015), anti-tumor (Lindholm et al., 2002; Du et al., 2020), immunosuppressive (Grundemann et al., 2013) as well as protease inhibition (Hellinger et al., 2015a).

Since proteases are interesting drug targets and many cystine-knot peptides have anti-protease activity, we were intrigued by studying Allexis cyclotides as protease inhibitors (Hellinger and Gruber, 2019). Momordica-type cyclotides (isolated from *Momordica cochinchinensis* (Lour.) Spreng. i.e. cyclic trypsin inhibitor peptides, MCoTI) are potent inhibitors of serine proteinases such as trypsin, chymotrypsin, matriptase, and beta-tryptase with pico- to nanomolar inhibitory potencies (Daly et al., 2013; Hellinger et al., 2015a; Hellinger and Gruber, 2019). Previous studies reported cyclotides from Psychotria plants (Rubiaceae) as inhibitors for the serine endopeptidase POP (Hellinger et al., 2015a). Since Allexis peptides have structural similarity to these previously characterized inhibitors, Allexis extracts were studied for inhibition of post-proline endopeptidase activity using the prototypic enzyme POP. All four Allexis extracts showed similar inhibitory action in the low microgram per milliliter range. Bioactivity-guided fractionation of plant extracts from *A. cauliflora* were performed with the aim to isolate bioactive plant peptides. The technique has been applied successfully in the past to identify and isolate bioactive cyclotides from plants (Tarrago et al., 2007; Hellinger et al., 2015a; Retzl et al., 2020). Several purification steps yielded two *A. cauliflora* peptides, denoted alca 1 and alca 2, which obtained an IC_50_ between 4 and 8 µM for POP. The calculated Hill factor of alca 1 is near unity and hence may be representative for a classical competitive inhibition mode. For alca 2 the Hill factor is slightly higher than 1; this is 1) indicative of a cooperative effect, such as exosite binding or allosteric modulation of enzymatic activity (Farady and Craik, 2010; Hellinger and Gruber, 2019), 2) it may be due to the complex kinetics of POP substrates/inhibitors (Mannisto and Garcia-Horsman, 2017), as alca 2 in low concentrations appears to activate POP, or 3) it may simply be an artefact, due to the lower purity of alca 2; this will have to be addressed in detailed mode of action studies of alca peptides and other cyclotides, in the future.

The novel peptides were further characterized with *de novo* amino acid sequencing to obtain their primary structure. Alca 1 and alca 2 comprise the six cysteines, a single glutamic acid and a head-to-tail cyclized peptide chain, which are characteristic elements of cyclotides. Both molecules were classified as bracelet type due to the absence of a *cis*-configured proline in loop 5 (Craik et al., 1999; Hellinger et al., 2015a). Thus, they are not only amongst the first identified cyclotides from *A. cauliflora* from the tribe Allexis of Violaceae (Craik et al., 1999), but alca peptides are new prototypic bracelet cyclotide inhibitors of human POP.

The overall similar inhibitory potencies of the reported isotypic cyclotide inhibitors may reflect a family wide conserved binding motif. Hence, we were interested to compare the sequences of bracelet and *Moebius* type cyclotides of Violaceae. A sequence logo was prepared to compare the amino acid positions for conserved residues or to identify variable positions (Figure 7C). Sequence homology in some segments of the molecule, e.g. the native ligation site (loop 6), as well as loops 1 and 4, is noted reflecting also conserved gene- and precursor structures (Park et al., 2017) (Supplementary Figure 10 **)**. The glutamic acid and other positional preserved residues are important for the intramolecular hydrogen bond network, which further stabilizes the CCK fold (Rosengren et al., 2003). Since loop 6 incorporates the native ligation site for backbone cyclization of cyclotides the conservation of residues (e.g. G, N, P) is highest, with the exception of acyclotides (Saska et al., 2007). Elevated amino acid diversity of some inter-cystine loops is noted comparing the two sub-types. Major differences were identified in loops 2, 3, and 5 (Rosengren et al., 2003). It is interesting that violaceous *Moebius* as well as bracelet cyclotides have one conserved proline in the ligation loop. In contrast, other prolines are less conserved, for instance the novel alca peptides comprise prolines in loops 2 and 6, whereas secondary amino acids are present in loops 3, 5 and 6 of *Moebius* type cyclotides. The homology analysis highlights the conserved motif in loop 6, but any proline in cyclotides, except the *cis*-Pro in loop 5, could be part of a possible binding site for human POP. However, in future studies it will be necessary to collect detailed information on the interaction of the cyclotide-protease complex to enable further molecular and mechanistic insight. As an example, the role of the active-site loop of the Cucurbitaceae-type inhibitor MCoTI-II has been previously examined in co-crystallization experiments with trypsin (Daly et al., 2013). The prime binding site near the P1 residue (lysine) in loop 1 of MCoTI-II was shown to occupy the substrate binding pocket of the enzyme. In parallel, a secondary binding was determined near to the active site of the protease, which was amenable for binding of the cyclic backbone (loop 6) probably further stabilizing the inhibitor backbone as well as the peptide-protease complex. Hence, cyclization might have created a second binding site with added value for a tight inhibitor-protease complex. To date, it has not been studied if cyclotide inhibitors of POP take advantage of the cyclic backbone similar as cyclic trypsin inhibitors, or if the ligation loop itself is the inhibition motif for protease. However, a cyclic backbone appeared not mandatory for inhibition of POP by the knottin peptide bevuTI-I, which showed overall very similar inhibition activity compared to cyclotide inhibitors (Retzl et al., 2020). Since cyclotides comprise up to three prolines it will be necessary to consider all of them for the identification of the putative binding site to POP. The present study provides a first glimpse on the possible sequence-activity relationship of cyclic cystine-rich POP inhibitors and our work may provide starting points for future structural and mechanistic studies.

Ethnomedicinal studies have shown beneficial effects of cyclotide expressing plants in few disease conditions with relationship to POP. For instance, African traditional medicine describes the therapeutic application of *Rinorea dentata* (P. Beauv.) Kuntze from the Violaceae toward neurodegenerative diseases (Sonibare and Ayoola, 2015). Similarly plant extracts of the cyclotide expressing species *V. odorata* led to antihypertensive effects through vasodilation of peripheral arterioles (Siddiqi et al., 2012). To make use of these beneficial effects it will be necessary to further investigate POP inhibition by purified peptides from these herbal medicines rather than using plant extracts. The pharmacological inhibition of POP activity is often referred to as promising therapeutic strategy because inhibitors have obtained effects in mouse models of cognitive deficits and neurodegenerative diseases, such as and Parkinson’s disease (Lawandi et al., 2010; López et al., 2013). Consequently, many efforts have been put into drug development of potent POP inhibitors. For instance, the compound JTP-4819 was studied in clinical phase 1 and 2 (Umemura et al., 1997). However, JTP-4819 and all other clinically tested candidates failed to succeed or provided ambiguous outcomes in their clinical phase and no POP inhibiting drug has yet approval by the authorities (López et al., 2013). A moderate activity of the studied cyclotides mitigates pharmacological application at this development stage especially since there are several small molecule inhibitors known with nanomolar potency. Therefore, the accumulated knowledge from medicinal development of peptidomimetic compounds, such as KYP-2047 or JTP-4819, could provide guidance for the design of more potent cyclotide-based POP inhibitors in the future. This could include the incorporation of reactive site probes (e.g. aldehyde, sulfonyl fluoride or nitrile group), which has been applied for dead-end inhibitors to yield increased inhibitor potency (Guardiola et al., 2018). Additionally, cyclotides may be beneficial as templates to design novel POP inhibitors, since cycloretro-inverso peptide inhibitors had higher inhibition constants for POP and negligible hydrolysis compared to the native cyclic or linear peptide (Gorrão et al., 2007). The CCK and other structural motifs in peptide-based protease inhibitors are stabilizing the molecular scaffolds and may be of advantage in terms of selectivity for a particular protease compared to protease isotypes (Hellinger and Gruber, 2019). Hence, it will be promising to utilize cyclotide scaffolds for POP drug development or as research probes (Craik and Du, 2017). This study shows that cyclotides are a novel class of POP inhibitors and our work may provide new drive for future development of peptide-based POP therapeutics. In summary, it will be of interest for the entire protease drug development and peptide field to further study cyclic cystine-rich peptide inhibitors of prototypic POP or other post-proline cleaving enzymes in the future.

This study reports the discovery of two novel bracelet type cyclotides from *A. cauliflora* with micromolar inhibitory activity for human POP. The herein established procedures on isolation, identification and bioactivity characterization might be applicable for research on other plant peptides. The new amino acid sequences highlight the diversity of plant-derived cyclic cystine-rich peptide to provide inhibitors of disease-relevant human proteases. The analysis of peptide sequences can be a starting point for a future study investigating the binding site for POP. It will especially be interesting to elucidate the role of the cyclic cystine-rich backbone of cyclotides in the interaction with the protease. This future work will also shed light on the mode of inhibition, e.g. does the peptide inhibitor bind in a strictly substrate-like manner or is the inhibition based on interactions with catalytic residues and/or additional secondary binding/allosteric sites.

## Data Availability

The original contributions presented in the study are publicly available. This data can be found here: The mass spectrometry MALDI-MS and MS/MS data have been deposited to the ProteomeXchange Consortium via the PRIDE partner repository with the dataset identifier PXD026664.
