# Supplementary material for: Cyclotides Isolated From Violet Plants of Cameroon Are Inhibitors of Human Prolyl Oligopeptidase
Source: Front Pharmacol. 2021 Jul 12;12:707596. doi: 10.3389/fphar.2021.707596 (PMC8311463; doi:10.3389/fphar.2021.707596)
Supplement: Supplementary file 1 [file DataSheet1.pdf]

Cyclotides isolated from Allexis species of Cameroon are inhibitors of human prolyl oligopeptidase

Jasmin Gattringer,[#] Olivier Eteme N'Dogo,[#] Bernhard Retzl, Carina Ebermann, Christian W. Gruber[*] and Roland Hellinger

Medical University of Vienna, Center for Physiology and Pharmacology, Schwarzspanierstr. 17, 1090 Vienna, Austria

[#] The authors contributed equally to the study.

[*]Corresponding author: Christian W. Gruber, Tel.:+43-(0)1-40160-31390, Email: christian.w.gruber@meduniwien.ac.at

Supplementary File

TABLE OF SUPPLEMENTARY DATA

Supplementary Figures S1-S9 .... S3

Table S1. Peptidomic analysis of Allexis batangae peptide enriched extracts........ S13
Table S2. Peptidomic analysis of Allexis obanensis peptide enriched extracts........ S13
Table S3. Peptidomic analysis of Allexis zygomorpha peptide enriched extract........ S14
Table S4. High sensitivity amino acid analysis of alca 1........ S15
Table S5. High sensitivity amino acid analysis of alca 2........ S15

Supplementary Data S1: Sequence alignment of cyclotides to alca 1 and alca 2 .... S16

SUPPLEMENTARY FIGURES

Supplementary Figure S1. Analysis of peptide masses in Allexis species. MALDI-TOF-MS spectra of peptide enriched extracts of A. batangae (A), A. obanensis (C) and A. zygomorpha (E) were analysed in the m/z range of 2900-3550. The two major signals were retrospectively denoted as alca 1 (m/z 3211.5) and alca 2 (m/z 3084.2) in the spectra. Representative pictures of each plant species are shown as an insert. Analysis of the extracts using analytical HPLC at absorbance at 280 nm are shown for A. batangae (B), A. obanensis (D) and A. zygomorpha (F).

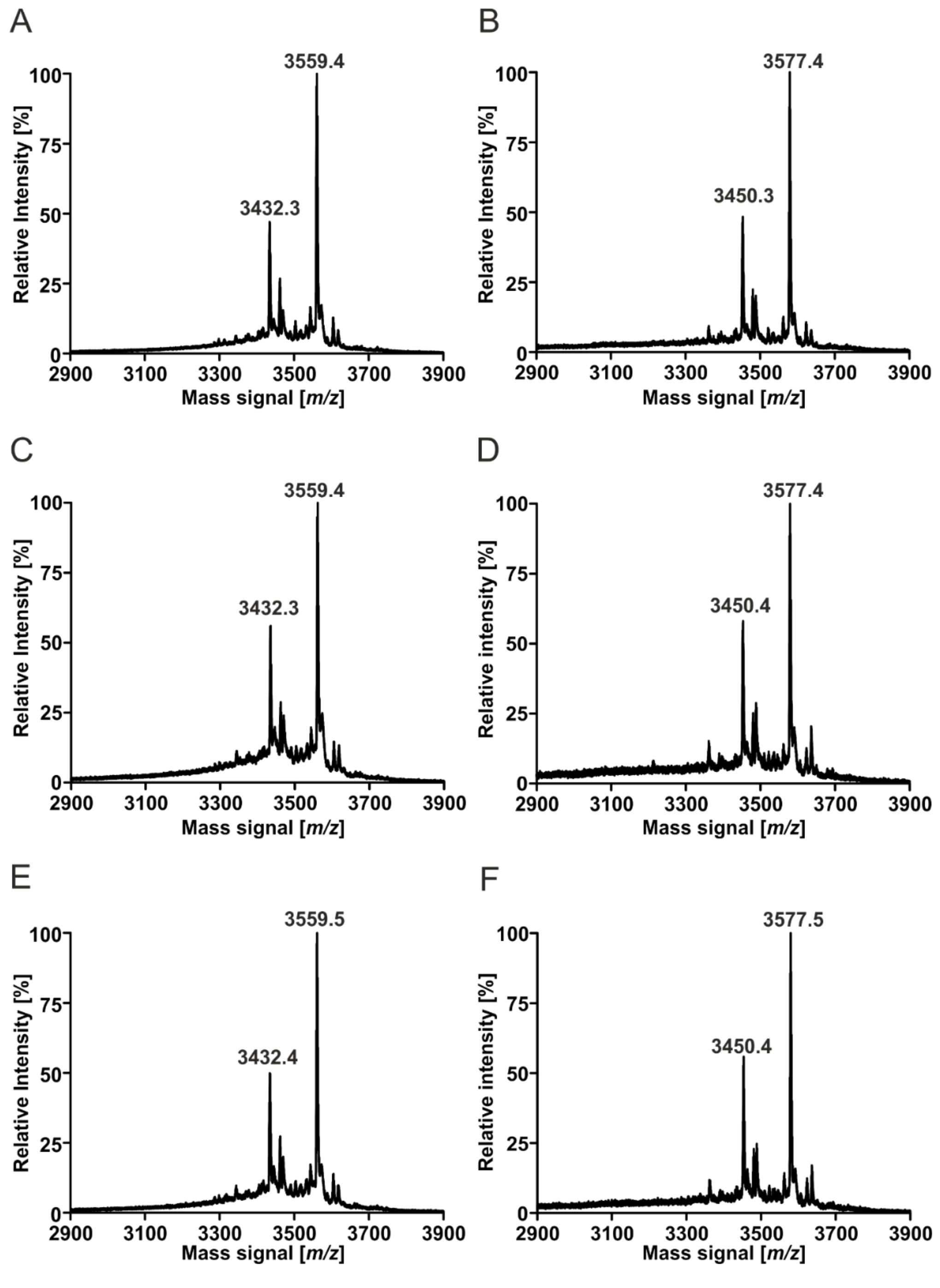

Supplementary Figure S2. Identification of cyclic cystine-rich peptides in Allexis species. Using a combined derivatization and site specific proteolysis strategy the number of cysteine moieties and the presence of a single conserved glutamic acid in the peptides of A. batangae (A, B), A. obanensis (C, D) and A. zygomorpha (F,G) were confirmed using MALDI-TOF-MS analysis of samples shown in the range of m/z 2900-3900. Characteristic mass shifts compared to the native mass were observed, such as +1 Da as well as of +58 Da per reduced and acetamidated cysteine, respectively, and additional +18 Da for a single peptide bond hydrolysis of endoprotease GluC activity. The total mass shift of +366 Da compared to the native mass indicates the presence of cyclic cystine-rich peptides in the investigated plant species. The observed masses of the two major peaks are labelled in each spectrum.

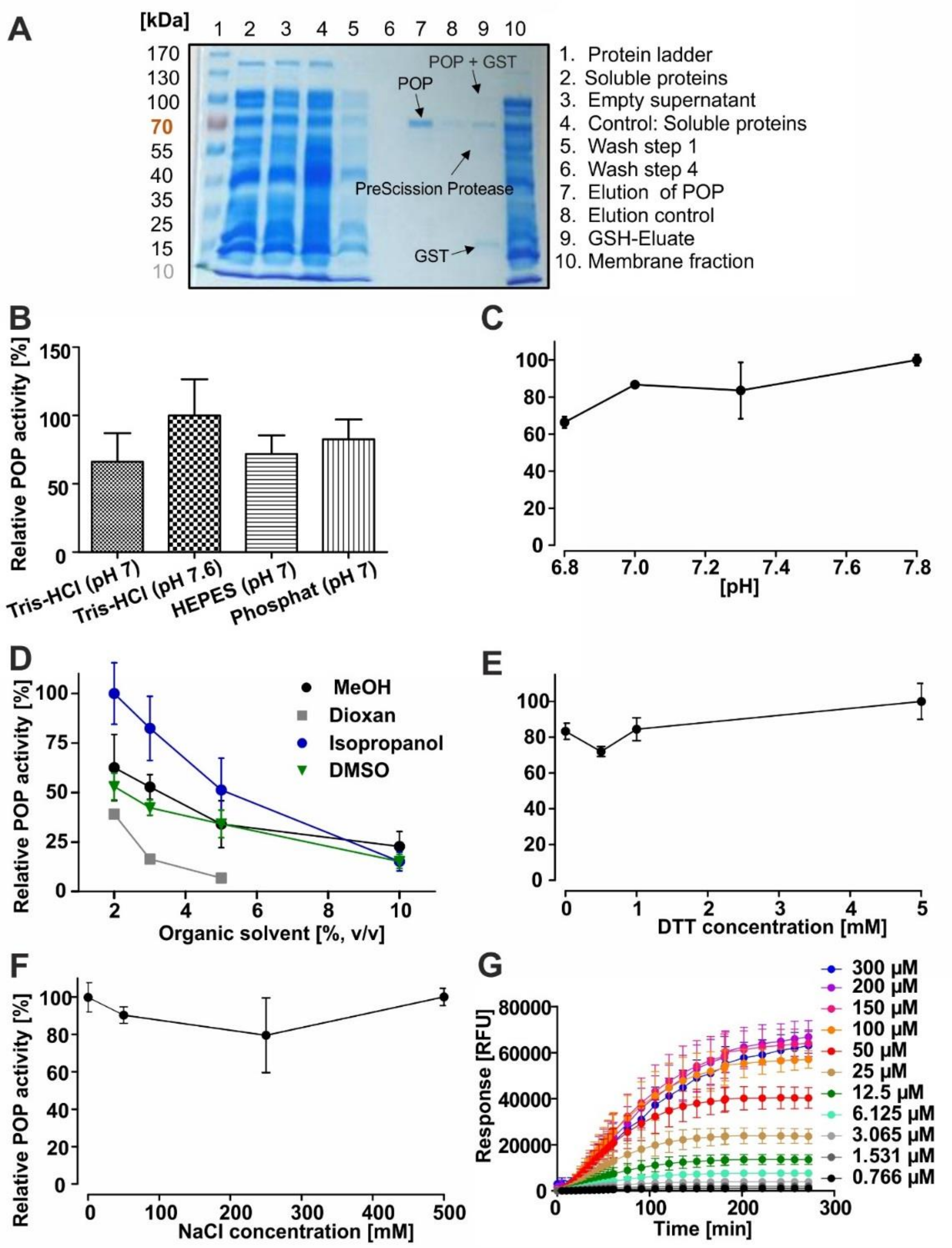

Supplementary Figure S3. POP purification and optimization of the assay system. Human POP was expressed in E. coli and purified as functional protease. The purification steps were analyzed using a Coomassie stained protein gel. Purified POP enzyme is shown in lane 7 after site-specific cleavage with PreScission protease and release of the untagged protein (A). Different assay conditions were tested to study the effect of buffer systems and pH dependency (B,C), the use of organic solvents (D), DTT (E) and NaCl (F) concentration on POP enzyme activity. For calculation of POP activity the slope in the linear range between 5 and 35 min of kinetic measurements were used. With the optimized conditions Z-Gly-Pro-amino-coumarin concentrations between 0.766 µM and 300 µM were tested in a kinetic measurement and a concentration dependent increase in signal was observed. Above 150 µM substrate concentration no further increase in signal was measured due to precipitation of the substrate (G). All data are shown as mean of three biological experiments and error is shown as ±SEM.

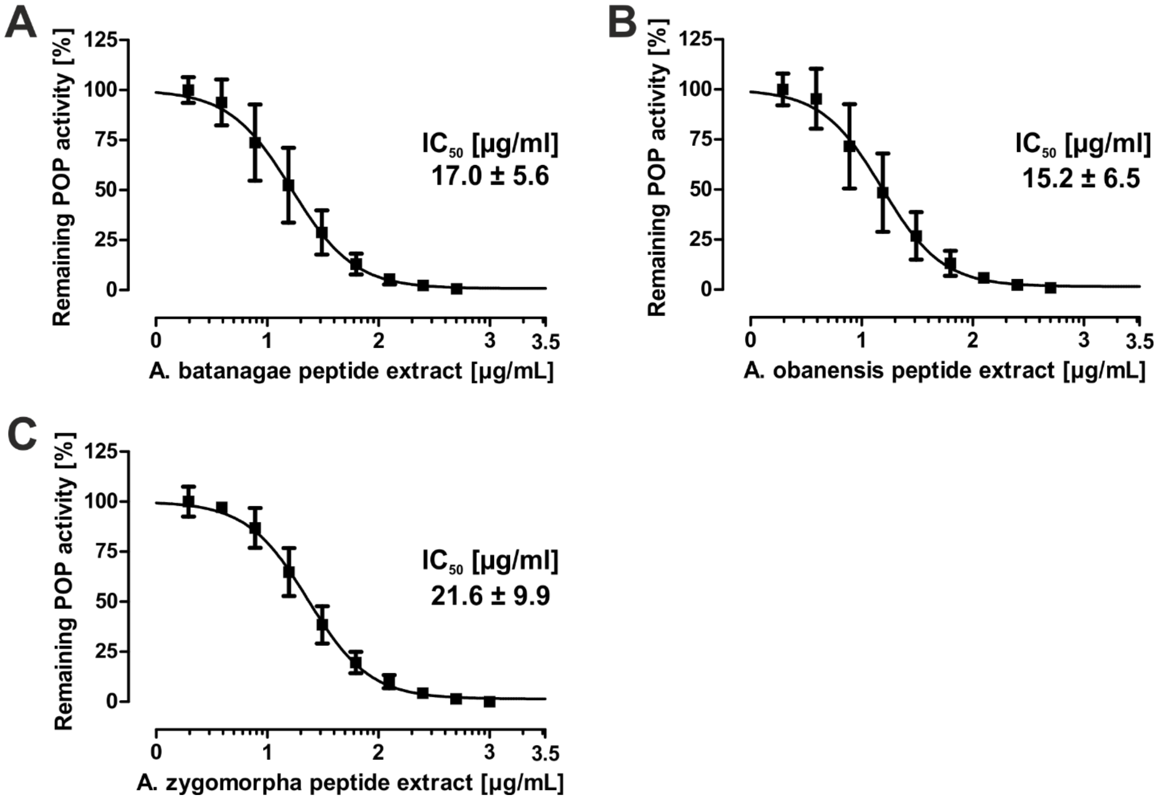

Supplementary Figure S4. POP inhibition of peptide enriched extracts of A. batangae, A. obanensis and A. zygomorpha. The inhibition activity of peptide enriched plant extracts toward human POP was evaluated in a dose response study applying 2-1000 µg/mL. The dose-response curves with the obtained IC_{50} values of the three plant species A. batangae (IC_{50} 17.0±5.6 µg/mL) (A), A. obanensis (IC_{50} 15.2±6.5) (B) and A. zygomorpha (IC_{50} 21.6±9.9 µg/mL) (C). All data are shown as n=6 including mean±standard deviation.

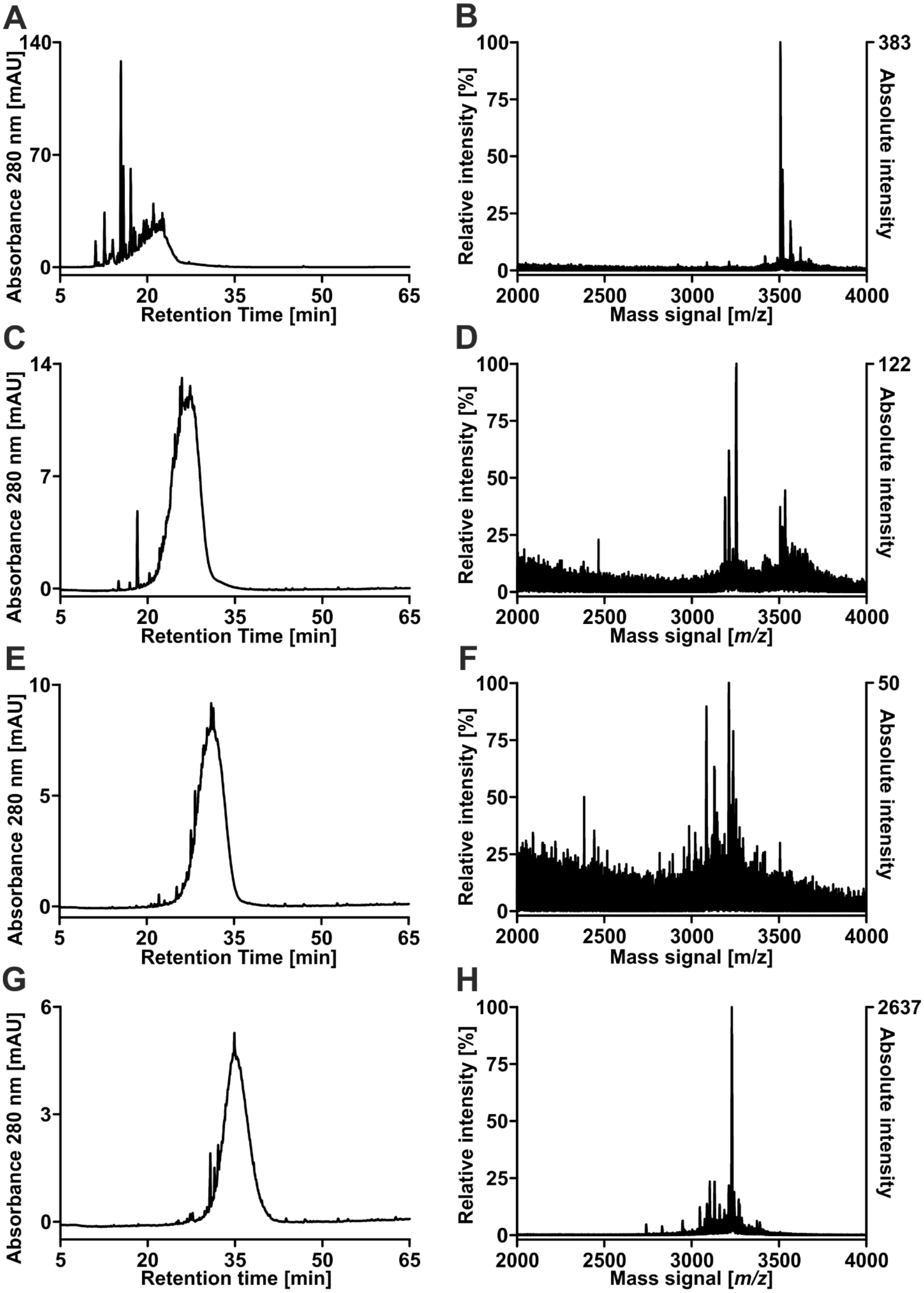

Supplementary Figure S5. QC of non-bioactive HPLC fractions. Analytical HPLC traces at 280 nm for non-bioactive fractions F2 (A), F3 (C), F4 (E) and F5 (G) and the corresponding MALDI-TOF-MS analysis in the range of m/z 2000-4000 for fractions F2 (B), F3 (D), F4 (F) and F5 (H).

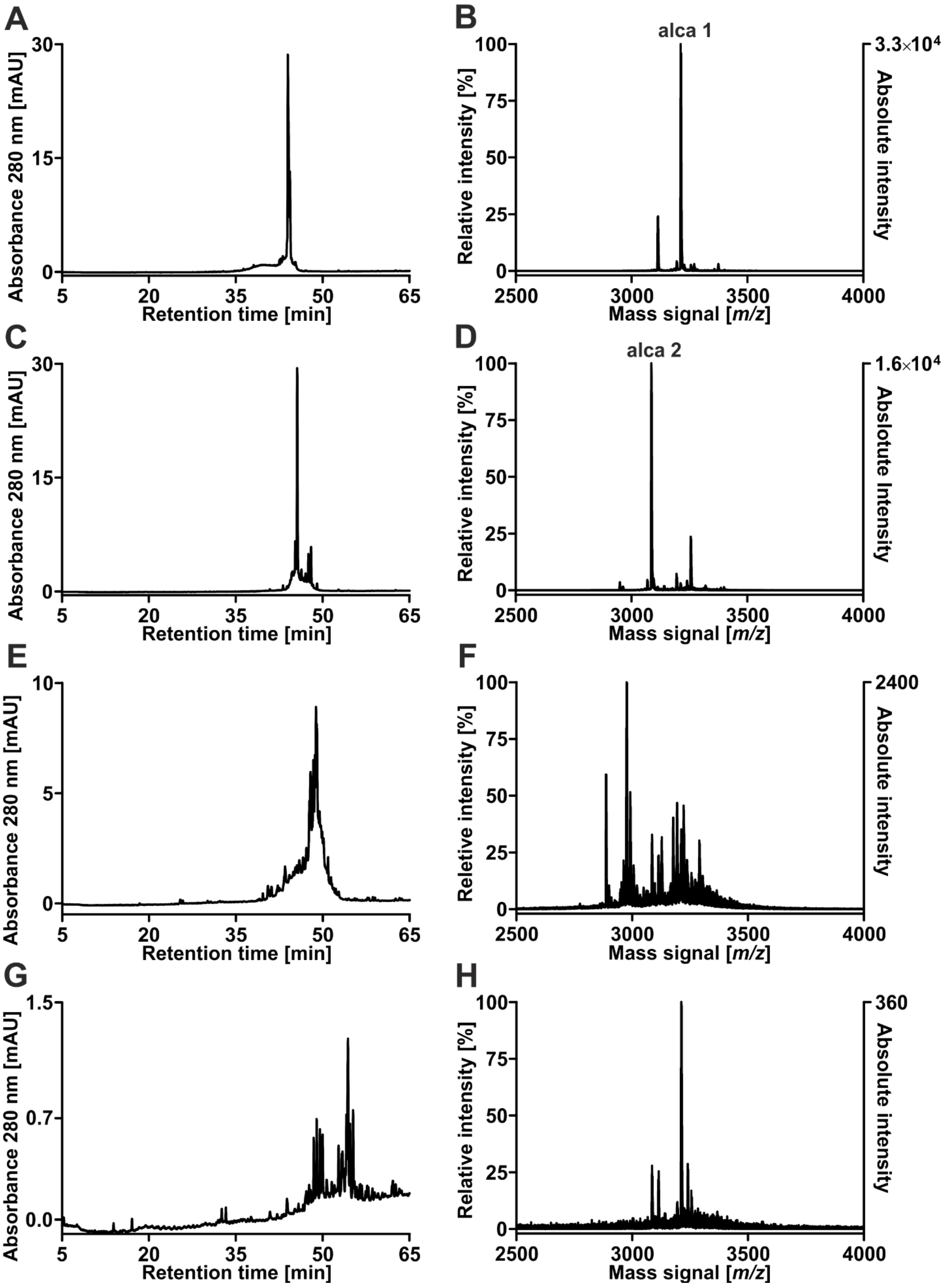

Supplementary Figure S6. The MALDI-MS and HPLC quality analysis are shown for the bioactive fractions derived from bioassay guided fraction. Analytical HPLC traces at 280 nm for bioactive fractions F6 (A), F7 (C), F8 (E) and F9 (G) and the corresponding MALDI-TOF-MS analysis in the range of m/z 2500-4000 for fractions F6 (B), F7 (D), F8 (F) and F9 (H). The two major peptides alca 1 (m/z 3211.5) and alca 2 (m/z 3084.2) identified in fractions F7 and F6 respectively were annotated in the spectra.

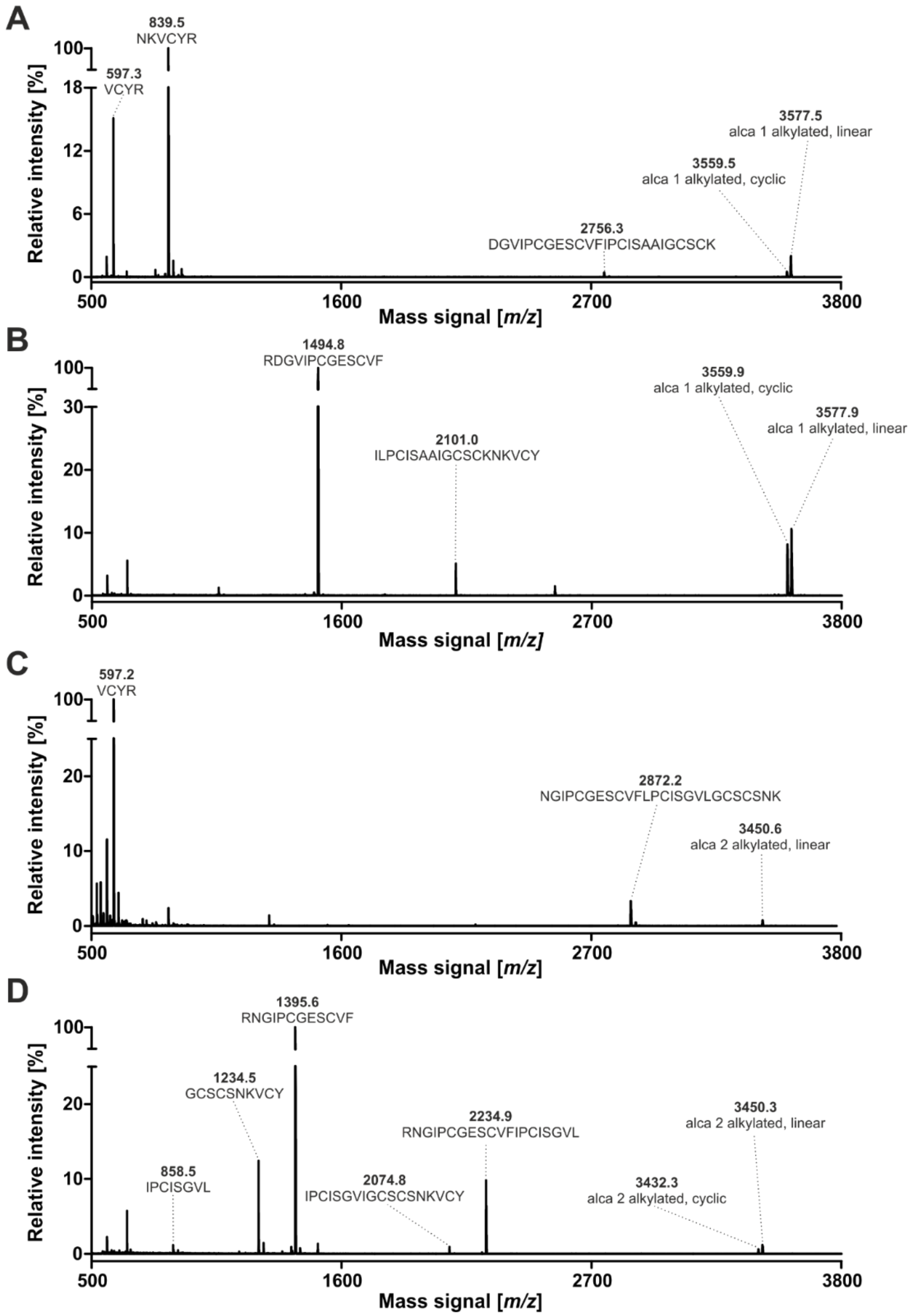

Supplementary Figure S7. Tryptic and chymotryptic fragment peptide analysis for alca 1 and alca 2. MALDI-MS analysis of tryptic and chymtotryptic digests of alca 1 (A, B) and alca 2 (C, D) are shown with the observed masses and the assigned amino acid sequences of the fragments. The alkylated, cyclic peptides and the respective linear peptides are labelled in the spectra where these peaks were visible. The cyclic peptide sequences are cyclo-GVIPCGESCVFIPCISAAIGCSCKNKVCYRD (alca 1) and cyclo-GIPCGESCVFIPCISGVLGCSCSNKVCYRN (alca 2). Tryptic digest was performed for 2.5 h at 37°C, chymotryptic fragments were measured after 1.5 h digestion time at RT. Spectra are shown in the mass range of m/z 500-3800.

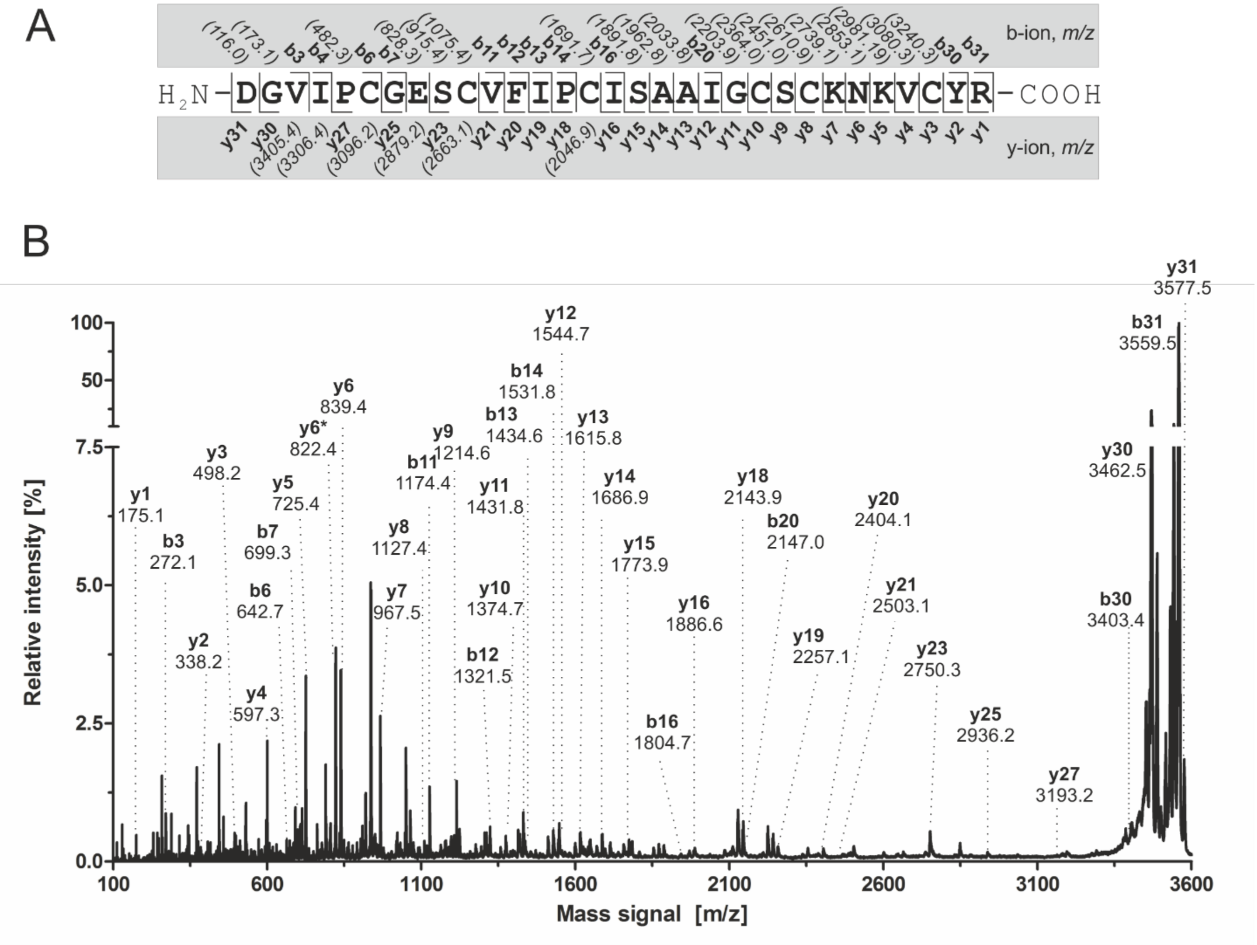

Supplementary Figure S8. Alca 1 fragmentation spectrum of tryptic digest. Annotation of tryptic full length precursor m/z 3577.5 of alca 1 (A). The annotated sequence is shown on the top. The b- and y-ions are assigned with corresponding mass signals. The annotated fragment ion spectrum provided (B). The asterisk indicates loss of ammonia (-17 Da).

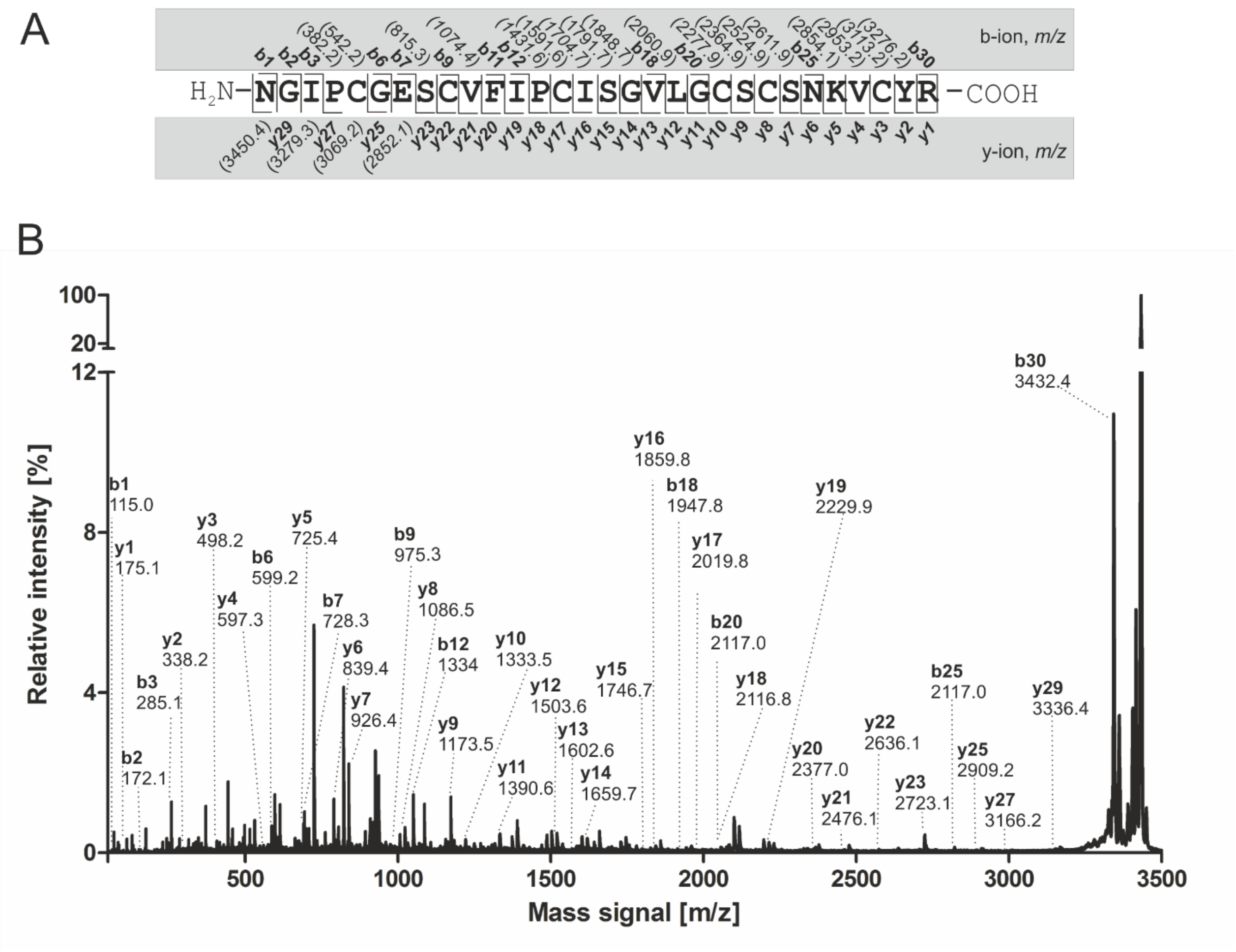

Supplementary Figure S9. Alca 2 fragmentation spectrum of tryptic digest Annotation of tryptic full length precursor m/z 3540.6 of alca 2 (A). The annotated sequence is shown on the top. The b- and y-ions are assigned with corresponding mass signals. The annotated fragment ion spectrum provided (B).

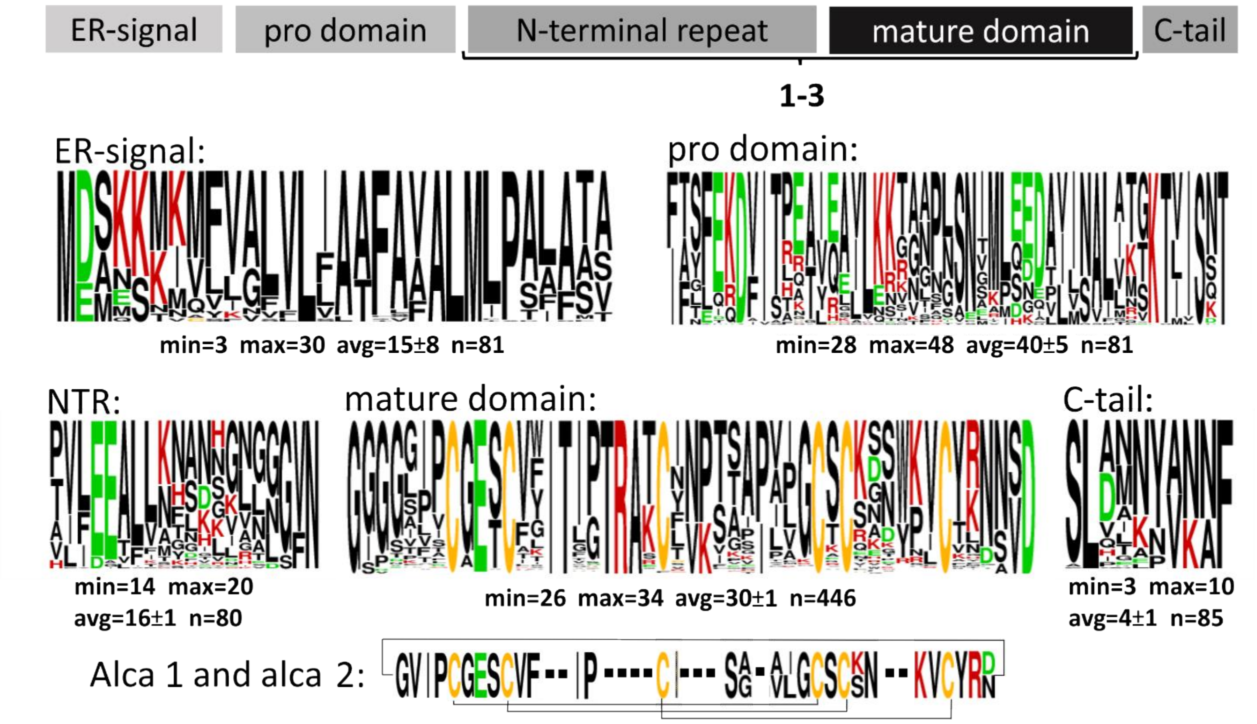

Supplementary Figure S10. Precursor analysis of violaceus cyclotides genes. Cyclotides are synthesized from precursor proteins typically consisting of an endoplasmic reticulum signal (ER-signal), a pro domain, a N-terminal repeat (NTR), a mature domain and a C-terminal tail (C-tail). The N-terminal repeat and the mature domain can be repeated up to three times. Annotated precursor proteins from Violaceae species were retrieved from Cybase and aligned with Clustal Omega. From all regions frequency plots were made with Weblogo and the minimum (min), maximum (max) and average (avg) length of each region ± standard deviation was calculated using custom Python scripts. Untypical sequences have been excluded from the analysis and the number (n) of used sequences is given. The frequency plot of alca 1 and alca 2 was aligned to the mature domain. Gaps (-) were introduced to maximize the alignment. Amino acids positively charged at pH7 (H, K, R) are coloured in red, amino acids negatively charged at pH7 (D, E) are coloured in green and cysteines (C) are shown in orange. The cyclic cystine knot topology is indicated in the frequency plot of alca 1 and alca 2.

Table S1. Peptidomic analysis of Allexis batangae peptide enriched extracts.

Native mass signal (m/z)[1]	Mass signal of cysteine reduced sample (m/z)[2]	Mass signal of cysteine carbamdiomethylated sample (m/z)[3]	Mass signal of EndoGluc processed (m/z)[4]
2948.2	2954.1	3296.3	3314.3
2962.3	2968.0	3310.2	3328.1
3057.2	3063.5	3405.1	3423.4
3067.2	3073.3	3415.3	3433.3
3084.1	3090.2	3432.3	3450.2
3111.2	3117.3	3459.4	3477.4
3193.1	3199.3	3541.5	3559.4
3210.3	3216.1	3558.4	3576.5
3254.4	3260.1	3602.4	3620.5
3268.2	3274.2	3616.5	3634.4
3372.3	3378.2	3720.5	3738.5
3398.3	3404.3	3746.5	3764.3

1 monoisotopic mass signals are shown
2 Full cysteine reduction with DTT obtains a mass shift of +1.0079 Da per residue
3 Carbamidomethylation with IAA obtains a mass shift of +348.1757 Da
4 Proteolytic cleavage of endoprotease GluC provides a mass shift of +18 Da corresponding to a conversion of a cyclic to a linear fragment peptide

Table S2. Peptidomic analysis of Allexis obanensis peptide enriched extracts.

**Native mass signal (m/z)[1]**	**Mass signal of cysteine reduced sample (m/z)[2]**	**Mass signal of cysteine carbamdiomethylated sample (m/z)[3]**	**Mass signal of EndoGluc processed (m/z)[4]**
3067.2	3073.4	3415.0	3433.3
3084.2	3090.0	3432.3	3450.2
3097.4	3103.0	3445.3	3463.1
3111.4	3117.1	3459.1	3477.0
3182.3	3188.4	3530.1	3548.0
3193.4	3199.4	3541.2	3559.6
3210.3	3216.0	3558.2	3576.4
3254.2	3260.1	3602.1	3620.0
3268.2	3274.1	3616.5	3634.2
3372.1	3378.2	3720.5	3738.6

1 monoisotopic mass signals are shown
2 Full cysteine reduction with DTT obtains a mass shift of +1.0079 Da per residue
3 Carbamidomethylation with IAA obtains a mass shift of +348.1757 Da
4 Proteolytic cleavage of endoprotease GluC provides a mass shift of +18 Da corresponding to a conversion of a cyclic to a linear fragment peptide

Table S3. Peptidomic analysis of Allexis zygomorpha peptide enriched extract.

Native mass signal (m/z)[1]	Mass signal of cysteine reduced sample (m/z)[2]	Mass signal of cysteine carbamdiomethylated sample (m/z)[3]	Mass signal of EndoGluc processed (m/z)[4]
2948.2	2954.3	3296.1	3314.1
2962.1	2968.2	3310.2	3328.2
3067.3	3073.1	3415.3	3433.3
3084.2	3090.1	3432.4	3450.3
3097.0	3103.3	3445.1	3463.4
3111.1	3117.0	3459.1	3477.4
3182.1	3188.0	3530.2	3548.5
3193.2	3199.0	3541.4	3559.5
3210.4	3216.1	3558.1	3576.5
3228.1	3234.2	3576.2	3594.3
3254.1	3260.2	3602.4	3620.5
3268.2	3274.2	3616.4	3634.2
3316.3	3322.5	3664.4	3682.1
3355.2	3361.4	3703.0	3721.5
3372.0	3378.1	3720.1	3738.6
3398.3	3404.3	3746.5	3764.5

1 monoisotopic mass signals are shown
2 Full cysteine reduction with DTT obtains a mass shift of +1.0079 Da per residue
3 Carbamidomethylation with IAA obtains a mass shift of +348.1757 Da
4 Proteolytic cleavage of endoprotease GluC provides a mass shift of +18 Da corresponding to a conversion of a cyclic to a linear fragment peptide

Table S4. High sensitivity amino acid analysis of alca 1

Amino acid**	ug / sample*	nmol / sample	Moles (%)	Number of amino acid residues
Serine	24.4	281	11.3	3
Arginine	16.6	107	4.3	1
Glycine	17.9	314	12.6	3
Aspartic acid#	23.8	206	8.3	2
Glutamic acid#	13.9	107	4.3	1
Alanine	13.9	196	7.9	2
Proline	20.5	211	8.5	2
Lysine	24.8	193	7.8	2
Tyrosine	16.8	103	4.1	1
Valine	28.2	284	11.4	3
Isoleucine	43.0	380	15.3	4
Phenylalanine	15.4	105	4.2	1
Total	259.1	2486	100.0	25

** cysteine and tryptophane residues were not analysed
* calculation based on amino acid residue mass (molecular weight minus H_2O), reporting limit was 0.8 µg/sample
# due to hydrolyzation Asp/Asn and Glu/Gln cannot be distinguished

Table S5. High sensitivity amino acid analysis of alca 2

Amino Acid**	ug / sample*	nmol / sample	Moles (%)	Number of amino acid residues
Serine	8.20	94.2	15.1	4
Arginine	4.13	26.4	4.2	1
Glycine	5.82	102.1	16.4	4
Aspartic acid#	6.09	52.9	8.5	2
Glutamic acid#	3.52	27.3	4.4	1
Proline	5.17	53.3	8.6	2
Lysine	3.53	27.6	4.4	1
Tyrosine	4.29	26.3	4.2	1
Valine	8.01	80.8	13.0	3
Isoleucine	8.89	78.6	12.6	3
Leucine	2.98	26.3	4.2	1
Phenylalanine	3.97	27.0	4.3	1
Total	64.60	622.6	100.0	24

** cysteine and tryptophane residues were not analysed
* calculation based on amino acid residue mass (molecular weight minus H_2O), reporting limit was 0.4 µg/sample
# due to hydrolyzation Asp/Asn and Glu/Gln cannot be distinguished

Supplementary Data S1: Sequence alignment of cyclotides to alca 1 and alca 2

Alignment of alca peptides to known cyclotide sequences obtained from CyBase was performed. The best 5 alignments are shown for alca 1 as well as alca 2.

alca 1

```
         alca 1     GVIPCGESCVFIPCISAAIGCSCKNKVCYRD-
                    ||||||||||||||||||||||||||||||
   Cycloviolin_A     GVIPCGESCVFIPCISAAIGCSCKNKVCYR-N


         alca 1     GVIPCGESCVFIPCIS-AAI-GCSCKNKVCYRD
                    |||||||||||||||  ||  ||||||||||||
         psyle_F     GVIPCGESCVFIPCI-TAA-VGCSCKNKVCYRD


         alca 1     GVI-PCGESCVFIPCISAAIGCSCKNKVCYRD-
                    |   |||||||||||||||||||||||||||
   cycloviolin_D     G--FPCGESCVFIPCISAAIGCSCKNKVCYR-N


         alca 1     GVIPCGESCVF-IPCISAAIGCSCKNKVCYRD-
                    | ||||||||  |||||||||||||||||||
cycloviolacin_O17     G-IPCGESCV-WIPCISAAIGCSCKNKVCYR-N


         alca 1     GVIPCGESCVFIPCISAA--IGCSCKNKVCYRD-
                    ||||||||||||||||    ||||||||||||
          Cter_A     GVIPCGESCVFIPCIS--TVIGCSCKNKVCYR-N
```

alca 2

```
         alca 2     GIPCGESCVFIPCISGVL-GCSC-SNKVCYRN
                    |||||||||||||||||  |||| | ||||||
          Hyfl_I     GIPCGESCVFIPCISGV-IGCSCKS-KVCYRN


         alca 2     G-IPCGESCVFIPCISG-VLGCSCS-NKVCYRN
                    | ||||||||||||||  ||||||  |||||||
           Mra23     GVIPCGESCVFIPCIS-SVLGCSC-KNKVCYRN


         alca 2     G-IPCGESCVFIPCISG-VLGCSC-SNKVCYRN
                    | ||||||||||||||  |||||| | ||||||
         Globa_B     GVIPCGESCVFIPCIS-AVLGCSCKS-KVCYRN


         alca 2     GIPCGESCVF-IPCISG-VLGCSCSNKVCYRN
                    |||||||||  |||||  ||||||||||||||
          hyen_O     GIPCGESCV-WIPCIS-SVLGCSCSNKVCYRN


         alca 2     GIPCGESC----VF-IPCISG-VLGCSCSNKVCYRN
                    ||||||||    |  |||||  ||||||||||||||
       hyen_A  Y     GIPCGESCGESCV-WIPCIS-SVLGCSCSNKVCYRN
```
